# Outcome patterns in COVID-ARDS compared to ARDS caused by other respiratory infections: an individual patient data analysis of six observational studies

**DOI:** 10.62675/2965-2774.20260182

**Published:** 2026-01-28

**Authors:** Paula J. van Oosten, Siebe G. Blok, Pien Swart, Luciano Cesar Pontes Azevedo, Giacomo Bellani, Michela Botta, Elisa Estenssoro, Eddy Fan, Juliana Carvalho Ferreira, John G. Laffey, Ignacio Martin-Loeches, Anna Motos, Tai Pham, Oscar Peñuelas, Marcus J. Schultz, Ary Serpa, Antoni Torres Marti, Anissa M. Tsonas, Frederique Paulus, David Michael Paul van Meenen, ERICC, Amade Martinez, Livia Leal, Antonio Jorge Pereira, Marcelo de Oliveira Maia, Josè Aires, Claudio Piras, Eliana Bernadete Caser, Cora Lavigne Moreira, Pablo Braga Gusman, Dyanne Moysés Dalcomune, Alexandre Guilherme Ribeiro de Carvalho, Louise Aline Romão Gondim, Lívia Mariane Castelo Branco Reis, Daniel da Cunha Ribeiro, Leonardo de Assis Simões, Rafaela Siqueira Campos, José Carlos Fernandez Versiani dos Anjos, Frederico Bruzzi Carvalho, Rossine Ambrosio Alves, Lilian Batista Nunes, Álvaro Réa-Neto, Mirella Cristine de Oliveira, Luana Tannous, Brenno Cardoso Gomes, Fernando Borges Rodriguez, Priscila Abelha, Marcelo E Lugarinho, Andre Japiassu, Hélder Konrad de Melo, Elton Afonso Lopes, Pedro Varaschin, Vicente Cés de Souza Dantas, Marcos Freitas Knibel, Micheli Ponte, Pedro Mendes de Azambuja Rodrigues, Rubens Carmo Costa, Felipe Saddy, Théia Forny Wanderley Castellões, Suzana Alves Silva, Luiz Antonio Gomes Osorio, Dora Mannarino, Rodolfo Espinoza, Cassia Righy, Marcio Soares, Jorge Salluh, Lilian Tanaka, Daniel Aragão, Maria Eduarda Tavares, Maura Goncalves Pereira Kehdi, Valéria Maria Campos Rezende, Roberto Carlos Cruz Carbonell, Cassiano Teixeira, Roselaine Pinheiro de Oliveira, Juçara Gasparetto Maccari, Priscylla Souza Castro, Paula Berto, Patricia Schwarz, André Peretti Torelly, Thiago Lisboa, Edison Moraes, Felipe Dal-Pizzol, Cristiane Tomasi Damiani, Cristiane Ritter, Juliana Carvalho Ferreira, Ramon Teixeira Costa, Pedro Caruso, Cristina Prata Amendola, Amanda Maria R R de Oliveira, Ulysses V A Silva, Luciana Coelho Sanches, Rosana D S Almeida, Luciano Cesar Azevedo, Marcelo Park, Guilherme Schettino, Murillo Santucci Assunção, Eliezer Silva, Carlos Eduardo Barboza, Antonio Paulo Nassar, Paulo Fernando G M Marzocchi Tierno, Luis Marcelo Malbouisson, Lucas Oliveira, Davi Cristovao, Manoel Leitão, Ênio Rego, Fernanda Eugênia Fernandes, Marcelo Luz Pereira Romano, Alexandre Biasi Cavalcanti, Dalton de Souza Barros, Érica Aranha Suzumura, Karla Loureiro Meira, Gustavo Affonso de Oliveira, Paula Menezes Luciano, Evelin Drociunas Pacheco, Bruno Franco Mazza, Flavia Ribeiro Machado, Elaine Ferreira, Ronaldo Batista dos Santos, Alexandra Siqueira Colombo, Antonio Carlos Nogueira, Juliana Baroni Fernandes, Raquel Siqueira Nóbrega, Barbara do CS Martins, Francisco Soriano, Rafaela Deczka Morsch, Andre Luiz Baptiston Nunes, Juliano Pinheiro de Almeida, Ludhmila Hajjar, Sílvia Moulin, Fábio Poianas Giannini, Andre Luiz Baptiston Nunes, LUNG SAFE, Fernando Rios, Frank Van Haren, T Sottiaux, Fredy S Lora, Luciano C Azevedo, P Depuydt, Eddy Fan, Guillermo Bugedo, Haibo Qiu, Marcos Gonzalez, Juan Silesky, Vladimir Cerny, Jonas Nielsen, Manuel Jibaja, Tài Pham, Hermann Wrigge, Dimitrios Matamis, Jorge Luis Ranero, S M Hashemian, Pravin Amin, Kevin Clarkson, Giacomo Bellani, Kiyoyasu Kurahashi, Asisclo Villagomez, Amine Ali Zeggwagh, Leo M Heunks, Jon Henrik Laake, Jose Emmanuel Palo, Antero do Vale Fernandes, Dorel Sandesc, Yaasen Arabi, Vesna Bumbasierevic, Jose A Lorente, Anders Larsson, Lise Piquilloud, Fekri Abroug, Daniel F McAuley, Lia McNamee, Javier Hurtado, Ed Bajwa, Gabriel Démpaire, Guy M Francois, Hektor Sula, Lordian Nunci, Alma Cani, Alan Zazu, Christian Dellera, Carolina S Insaurralde, Risso V Alejandro, Julio Daldin, Mauricio Vinzio, Ruben O Fernandez, Luis P Cardonnet, Lisandro R Bettini, Mariano Carboni Bisso, Emilio M Osman, Mariano G Setten, Pablo Lovazzano, Javier Alvarez, Veronica Villar, Cesar Milstein, Norberto C Pozo, Nicolas Grubissich, Gustavo A Plotnikow, Daniela N Vasquez, Santiago Ilutovich, Norberto Tiribelli, Ariel Chena, Carlos A Pellegrini, María G Saenz, Elisa Estenssoro, Matias Brizuela, Hernan Gianinetto, Pablo E Gomez, Valeria I Cerrato, Marco G Bezzi, Silvina A Borello, Flavia A Loiacono, Adriana M Fernandez, Serena Knowles, Claire Reynolds, Deborah M Inskip, Jennene J Miller, Jing Kong, Christina Whitehead, Shailesh Bihari, Aylin Seven, Amanda Krstevski, Helen J Rodgers, Rebecca T Millar, Toni E Mckenna, Irene M Bailey, Gabrielle C Hanlon, Anders Aneman, Joan M Lynch, Raman Azad, John Neal, Paul W Woods, Brigit L Roberts, Mark R Kol, Helen S Wong, Katharina C Riss, Thomas Staudinger, Xavier Wittebole, Caroline Berghe, Pierre A Bulpa, Alain M Dive, Rik Verstraete, Herve Lebbinck, Pieter Depuydt, Joris Vermassen, Philippe Meersseman, Helga Ceunen, Jonas I Rosa, Daniel O Beraldo, Claudio Piras, Adenilton M R Ampinelli, Antonio P Nassar, Sergio Mataloun, Marcelo Moock, Marlus M Thompson, Claudio H Gonçalves, Ana Carolina P Antônio, Aline Ascoli, Rodrigo S Biondi, Danielle C Fontenele, Danielle Nobrega, Vanessa M Sales, Suresh Shindhe, Dk Maizatul Aiman B Pg Hj Ismail, John Laffey, Francois Beloncle, Kyle G Davies, Rob Cirone, Venika Manoharan, Mehvish Ismail, Ewan C Goligher, Mandeep Jassal, Erin Nishikawa, Areej Javeed, Gerard Curley, Nuttapol Rittayamai, Matteo Parotto, Niall D Ferguson, Sangeeta Mehta, Jenny Knoll, Antoine Pronovost, Sergio Canestrini, Alejandro R Bruhn, Patricio H Garcia, Felipe A Aliaga, Pamela A Farías, Jacob S Yumha, Claudia A Ortiz, Javier E Salas, Alejandro A Saez, Luis D Vega, Eduardo F Labarca, Felipe T Martinez, Nicolás G Carreño, Pilar Lora, Haitao Liu, Haibo Qiu, Ling Liu, Rui Tang, Xiaoming Luo, Youzhong An, Huiying Zhao, Yan Gao, Zhe Zhai, Zheng L Ye, Wei Wang, Wenwen Li, Qingdong Li, Ruiqiang Zheng, Wenkui Yu, Juanhong Shen, Xinyu Li, Tao Yu, Weihua Lu, Ya Q Wu, Xiao B Huang, Zhenyang He, Yuanhua Lu, Hui Han, Fan Zhang, Renhua Sun, Hua X Wang, Shu H Qin, Bao H Zhu, Jun Zhao, Jian Liu, Bin Li, Jing L Liu, Fa C Zhou, Qiong J Li, Xing Y Zhang, Zhou Li-Xin, Qiang Xin-Hua, Liangyan Jiang, Yuan N Gao, Xian Y Zhao, Yuan Y Li, Xiao L Li, Chunting Wang, Qingchun Yao, Rongguo Yu, Kai Chen, Huanzhang Shao, Bingyu Qin, Qing Q Huang, Wei H Zhu, Ai Y Hang, Ma X Hua, Yimin Li, Yonghao Xu, Yu D Di, Long L Ling, Tie H Qin, Shou H Wang, Junping Qin, Yi Han, Suming Zhou, Monica P Vargas, Juan I Silesky Jimenez, Manuel A González Rojas, Jaime E Solis-Quesada, Christian M Ramirez-Alfaro, Jan Máca, Peter Sklienka, Jakob Gjedsted, Aage Christiansen, Jonas Nielsen, Boris G Villamagua, Miguel Llano, Philippe Burtin, Gautier Buzancais, Pascal Beuret, Nicolas Pelletier, Satar Mortaza, Alain Mercat, Jonathan Chelly, Sébastien Jochmans, Nicolas Terzi, Cédric Daubin, Guillaume Carteaux, Nicolas de Prost, Jean-Daniel Chiche, Fabrice Daviaud, Tai Pham, Muriel Fartoukh, Guillaume Barberet, Jerome Biehler, Jean Dellamonica, Denis Doyen, Jean-Michel Arnal, Anais Briquet, Sami Hraiech, Laurent Papazian, Arnaud Follin, Damien Roux, Jonathan Messika, Evangelos Kalaitzis, Laurence Dangers, Alain Combes, Siu-Ming Au, Gaetan Béduneau, Dorothée Carpentier, Elie H Zogheib, Herve Dupont, Sylvie Ricome, Francesco L Santoli, Sebastien L Besset, Philippe Michel, Bruno Gelée, Pierre-Eric Danin, Bernard Goubaux, Philippe J Crova, Nga T Phan, Frantz Berkelmans, Julio C Badie, Romain Tapponnier, Josette Gally, Samy Khebbeb, Jean-Etienne Herbrecht, Francis Schneider, Pierre-Louis M Declercq, Jean-Philippe Rigaud, Jacques Duranteau, Anatole Harrois, Russell Chabanne, Julien Marin, Charlene Bigot, Sandrine Thibault, Mohammed Ghazi, Messabi Boukhazna, Salem Ould Zein, Jack R Richecoeur, Daniele M Combaux, Fabien Grelon, Charlene Le Moal, Elise P Sauvadet, Adrien Robine, Virginie Lemiale, Danielle Reuter, Martin Dres, Alexandre Demoule, Dany Goldgran-Toledano, Loredana Baboi, Claude Guérin, Ralph Lohner, Jens Kraßler, Susanne Schäfer, Kai D Zacharowski, Patrick Meybohm, Andreas W Reske, Philipp Simon, Hans-Bernd F Hopf, Michael Schuetz, Thomas Baltus, Metaxia N Papanikolaou, Theonymfi G Papavasilopoulou, Giannis A Zacharas, Vasilis Ourailogloy, Eleni K Mouloudi, Eleni V Massa, Eva O Nagy, Electra E Stamou, Ellada V Kiourtzieva, Marina A Oikonomou, Luis E Avila, Cesar A Cortez, Johanna E Citalán, Sameer A Jog, Safal D Sable, Bhagyesh Shah, Mohan Gurjar, Arvind K Baronia, Mohammedfaruk Memon, Radhakrishnan Muthuchellappan, Venkatapura J Ramesh, Anitha Shenoy, Ramesh Unnikrishnan, Subhal B Dixit, Rachana V Rhayakar, Nagarajan Ramakrishnan, Vallish K Bhardwaj, Heera L Mahto, Sudha V Sagar, Vijayanand Palaniswamy, Deeban Ganesan, Seyed Mohammadreza Hashemian, Hamidreza Jamaati, Farshad Heidari, Edel A Meaney, Alistair Nichol, Karl M Knapman, Donall O’Croinin, Eimhin S Dunne, Dorothy M Breen, Kevin P Clarkson, Rola F Jaafar, Rory Dwyer, Fahd Amir, Olaitan O Ajetunmobi, Aogan C O’Muircheartaigh, Colin S Black, Nuala Treanor, Daniel V Collins, Wahid Altaf, Gianluca Zani, Maurizio Fusari, Savino Spadaro, Carlo A Volta, Romano Graziani, Barbara Brunettini, Salvatore Palmese, Paolo Formenti, Michele Umbrello, Andrea Lombardo, Elisabetta Pecci, Marco Botteri, Monica Savioli, Alessandro Protti, Alessia Mattei, Lorenzo Schiavoni, Andrea Tinnirello, Manuel Todeschini, Antonino Giarratano, Andrea Cortegiani, Sara Sher, Anna Rossi, Massimo M Antonelli, Luca M Montini, Paolo Casalena, Sergio Scafetti, Giovanna Panarello, Giovanna Occhipinti, Nicolò Patroniti, Matteo Pozzi, Roberto R Biscione, Michela M Poli, Ferdinando Raimondi, Daniela Albiero, Giulia Crapelli, Eduardo Beck, Vincenzo Pota, Vincenzo Schiavone, Alexandre Molin, Fabio Tarantino, Giacomo Monti, Elena Frati, Lucia Mirabella, Gilda Cinnella, Tommaso Fossali, Riccardo Colombo, Pierpaolo Terragni, Ilaria Pattarino, Francesco Mojoli, Antonio Braschi, Erika E Borotto, Andrea N Cracchiolo, Daniela M Palma, Francesco Raponi, Giuseppe Foti, Ettore R Vascotto, Andrea Coppadoro, Luca Brazzi, Leda Floris, Giorgio A Iotti, Aaron Venti, Osamu Yamaguchi, Shunsuke Takagi, Hiroki N Maeyama, Eizo Watanabe, Yoshihiro Yamaji, Kazuyoshi Shimizu, Kyoko Shiozaki, Satoru Futami, Sekine Ryosuke, Koji Saito, Yoshinobu Kameyama, Keiko Ueno, Masayo Izawa, Nao Okuda, Hiroyuki Suzuki, Tomofumi Harasawa, Michitaka Nasu, Tadaaki Takada, Fumihito Ito, Shin Nunomiya, Kansuke Koyama, Toshikazu Abe, Kohkichi Andoh, Kohei Kusumoto, Akira Hirata, Akihiro Takaba, Hiroyasu Kimura, Shuhei Matsumoto, Ushio Higashijima, Hiroyuki Honda, Nobumasa Aoki, Hiroshi Imai, Yasuaki Ogino, Ichiko Mizuguchi, Kazuya Ichikado, Kenichi Nitta, Katsunori Mochizuki, Tomoaki Hashida, Hiroyuki Tanaka, Tomoyuki Nakamura, Daisuke Niimi, Takeshi Ueda, Yozo Kashiwa, Akinori Uchiyama, Olegs Sabelnikovs, Peteris Oss, Youssef Haddad, Kong Y Liew, Silvio A Ñamendys-Silva, Yves D Jarquin-Badiola, Luis A Sanchez-Hurtado, Saira S Gomez-Flores, Maria C Marin, Asisclo J Villagomez, Jordana S Lemus, Jonathan M Fierro, Mavy Ramirez Cervantes, Francisco Javier Flores Mejia, Daniel R Gonzalez, Dulce M Dector, Claudia R Estrella, Jorge R Sanchez-Medina, Alvaro Ramirez-Gutierrez, Fernando G George, Janet S Aguirre, Juan A Buensuseso, Manuel Poblano, Tarek Dendane, Amine Ali Zeggwagh, Hicham Balkhi, Mina Elkhayari, Nacer Samkaoui, Hanane Ezzouine, Abdellatif Benslama, Mourad Amor, Wajdi Maazouzi, Nedim Cimic, Oliver Beck, Monique M Bruns, Jeroen A Schouten, Myra Rinia, Monique Raaijmakers, Leo M Heunks, Hellen M Van Wezel, Serge J Heines, Marc P Buise, Fabienne D Simonis, Marcus J Schultz, Jennifer C Goodson, Troy S B rowne, Leanlove Navarra, Anna Hunt, Robyn A Hutchison, Mathew B Bailey, Lynette Newby, Colin Mcarthur, Michael Kalkoff, Alex Mcleod, Jonathan Casement, Danielle J Hacking, Finn H Andersen, Merete S Dolva, Jon H Laake, Andreas Barratt-Due, Kim Andre L Noremark, Eldar Søreide, Brit Å Sjøbø, Anne B Guttormsen, Hector H Leon Yoshido, Ronald Zumaran Aguilar, Fredy A Montes Oscanoa, Alain U Alisasis, Joanne B Robles, Rossini Abbie B Pasanting-Lim, Beatriz C Tan, Pawel Andruszkiewicz, Karina Jakubowska, Cristina M Cox, António M Alvarez, Bruno S Oliveira, Gustavo M Montanha, Nelson C Barros, Carlos S Pereira, António M Messias, Jorge M Monteiro, Ana M Araujo, Nuno T Catorze, Susan M Marum, Maria J Bouw, Rui M Gomes, Vania A Brito, Silvia Castro, Joana M Estilita, Filipa M Barros, Isabel M Serra, Aurelia M Martinho, Dana R Tomescu, Alexandra Marcu, Ovidiu H Bedreag, Marius Papurica, Dan E Corneci, Silvius Ioan Negoita, Evgeny Grigoriev, Alexey I Gritsan, Andrey A Gazenkampf, Ghaleb Almekhlafi, Mohamad M Albarrak, Ghanem M Mustafa, Khalid A Maghrabi, Nawal Salahuddin, Tharwat M Aisa, Ahmed S Al Jabbary, Edgardo Tabhan, Yaseen M Arabi, Olivia A Trinidad, Hasan M Al Dorzi, Edgardo E Tabhan, Stefan Bolon, Oliver Smith, Jordi Mancebo, Hernan Aguirre-Bermeo, Juan C Lopez-Delgado, Francisco Esteve, Gemma Rialp, Catalina Forteza, Candelaria De Haro, Antonio Artigas, Guillermo M Albaiceta, Sara De Cima-Iglesias, Leticia Seoane-Quiroga, Alexandra Ceniceros-Barros, Antonio L Ruiz-Aguilar, Luis M Claraco-Vega, Juan Alfonso Soler, Maria del Carmen Lorente, Cecilia Hermosa, Federico Gordo, Miryam Prieto-González, Juan B López-Messa, Manuel P Perez, Cesar P Pere, Raquel Montoiro Allue, Ferran Roche-Campo, Marcos Ibañez-Santacruz, Susana Temprano, Maria C Pintado, Raul De Pablo, Pilar Ricart Aroa Gómez, Silvia Rodriguez Ruiz, Silvia Iglesias Moles, Mª Teresa Jurado, Alfons Arizmendi, Enrique A Piacentini, Nieves Franco, Teresa Honrubia, Meisy Perez Cheng, Elena Perez Losada, Javier Blanco, Luis J Yuste, Cecilia Carbayo-Gorriz, Francisca G Cazorla-Barranquero, Javier G Alonso, Rosa S Alda, Ángela Algaba, Gonzalo Navarro, Enrique Cereijo, Esther Diaz-Rodriguez, Diego Pastor Marcos, Laura Alvarez Montero, Luis Herrera Para, Roberto Jimenez Sanchez, Miguel Angel Blasco Navalpotro, Ricardo Diaz Abad, Raquel Montiel González, Dácil Parrilla Toribio, Alejandro G Castro, Maria Jose D Artiga, Oscar Penuelas, Tomas P Roser, Moreno F Olga, Elena Gallego Curto, Rocío Manzano Sánchez, Vallverdu P Imma, Garcia M Elisabet, Laura Claverias, Monica Magret, Ana M Pellicer, Lucia L Rodriguez, Jesús Sánchez-Ballesteros, Ángela González-Salamanca, Antonio G Jimenez, Francisco P Huerta, Juan Carlos J Sotillo Diaz, Esther Bermejo Lopez, David D Llinares Moya, Alec A Tallet Alfonso, Palazon Sanchez Eugenio Luis, Palazon Sanchez Cesar, Sánchez I Rafael, Corcoles G Virgilio, Noelia N Recio, Richard O Adamsson, Christian C Rylander, Bernhard Holzgraefe, Lars M Broman, Joanna Wessbergh, Linnea Persson, Fredrik Schiöler, Hans Kedelv, Anna Oscarsson Tibblin, Henrik Appelberg, Lars Hedlund, Johan Helleberg, Karin E Eriksson, Rita Glietsch, Niklas Larsson, Ingela Nygren, Silvia L Nunes, Anna-Karin Morin, Thomas Kander, Anne Adolfsson, Lise Piquilloud, Hervé O Zender, Corinne Leemann-Refondini, Souheil Elatrous, Slaheddine Bouchoucha, Imed Chouchene, Islem Ouanes, Asma Ben Souissi, Salma Kamoun, Oktay Demirkiran, Mustafa Aker, Emre Erbabacan, Ilkay Ceylan, Nermin Kelebek Girgin, Menekse Ozcelik, Necmettin Ünal, Basak Ceyda Meco, Onat O Akyol, Suleyman S Derman, Barry Kennedy, Ken Parhar, Latha Srinivasa, Lia McNamee, Danny McAuley, Jack Steinberg, Phil Hopkins, Clare Mellis, Frank Stansil, Vivek Kakar, Dan Hadfield, Christine Brown, Andre Vercueil, Kaushik Bhowmick, Sally K Humphreys, Andrew Ferguson, Raymond Mckee, Ashok S Raj, Danielle A Fawkes, Philip Watt, Linda Twohey, Rajeev R Jha Matthew Thomas, Alex Morton, Varsha Kadaba, Mark J Smith, Anil P Hormis, Santhana G Kannan, Miriam Namih, Henrik Reschreiter, Julie Camsooksai, Alek Kumar, Szabolcs Rugonfalvi, Christopher Nutt, Orla Oneill, Colette Seasman, Ged Dempsey, Christopher J Scott, Helen E Ellis, Stuart Mckechnie, Paula J Hutton, Nora N Di Tomasso, Michela N Vitale, Ruth O Griffin, Michael N Dean, Julius H Cranshaw, Emma L Willett, Nicholas Ioannou, Sarah Gillis, Peter Csabi, Rosaleen Macfadyen, Heidi Dawson, Pieter D Preez, Alexandra J Williams, Owen Boyd, Laura Ortiz-Ruiz De Gordoa, Jon Bramall, Sophie Symmonds, Simon K Chau, Tim Wenham, Tamas Szakmany, Piroska Toth-Tarsoly, Katie H Mccalman, Peter Alexander, Lorraine Stephenson, Thomas Collyer, Rhiannon Chapman, Raphael Cooper, Russell M Allan, Malcolm Sim, David W Wrathall, Donald A Irvine, Kim S Zantua, John C Adams, Andrew J Burtenshaw, Gareth P Sellors, Ingeborg D Welters, Karen E Williams, Robert J Hessell, Matthew G Oldroyd, Ceri E Battle, Suresh Pillai, Istvan Kajtor, Mageswaran Sivashanmugave, Sinead C Okane, Adrian Donnelly, Aniko D Frigyik, Jon P Careless, Martin M May, Richard Stewart, T John Trinder, Samantha J Hagan, Matt P Wise, Jade M Cole, Caroline C MacFie, Anna T Dowling, Javier Hurtado, Nicolás Nin, Javier Hurtado, Edgardo Nuñez, Gustavo Pittini, Ruben Rodriguez, María C Imperio, Cristina Santos, Ana G França, Alejandro Ebeid, Alberto Deicas, Carolina Serra, Aditya Uppalapati, Ghassan Kamel, Valerie M Banner-Goodspeed, Jeremy R Beitler, Satyanarayana Reddy Mukkera, Shreedhar Kulkarni, Jarone Lee, Tomaz Mesar, John O Shinn Iii, Dina Gomaa, Christopher Tainter, Tomaz Mesar, R Adams Cowley, Dale J Yeatts, Jessica Warren, Michael J Lanspa, Russel R Miller, Colin K Grissom, Samuel M Brown, Philippe R Bauer, Ryan J Gosselin, Barrett T Kitch, Jason E Cohen, Scott H Beegle, Renaud M Gueret, Aiman Tulaimat, Shazia Choudry, William Stigler, Hitesh Batra, Nidhi G Huff, Keith D Lamb, Trevor W Oetting, Nicholas M Mohr, Claine Judy, Shigeki Saito, Fayez M Kheir, Adam B Schlichting, Angela Delsing, Mary Elmasri, Daniel R Crouch, Dina Ismail, Thomas C Blakeman, Kyle R Dreyer, Dina Gomaa, Rebecca M Baron, Carolina Quintana Grijalba, Peter C Hou, Raghu Seethala, Imo Aisiku, Galen Henderson, Gyorgy Frendl, Sen-Kuang Hou, Robert L Owens, Ashley Schomer, Vesna Bumbasirevic, Bojan Jovanovic, Maja Surbatovic, Milic Veljovic, PRoVENT–COVID, Jesse P. van Akkeren, Anna Geke Algera, Cheetel K. Algoe, Rombout B. van Amstel, Onno L. Baur, Pablo van de Berg, Alida E. van den Berg, Dennis C.J.J. Bergmans, Dido I. van den Bersselaar, Freke A. Bertens, Alexander J.G.H. Bindels, Siebe G. Blok, Milou M. de Boer, Sylvia den Boer, Leonoor S. Boers, Margriet Bogerd, Lieuwe D.J. Bos, Michela Botta, Jennifer S. Breel, Hendrik de Bruin, Sanne de Bruin, Caro L. Bruna, Laura A. Buiteman-Kruizinga, Olaf L. Cremer, Rogier M. Determann, Willem Dieperink, Dave A. Dongelmans, Hildegard S. Franke, Michal S. Galek-Aldridge, Mart J. de Graaff, Laura A. Hagens, Jasper J. Haringman, Sebastiaan T. van der Heide, Pim L.J. van der Heiden, Nanon F.L. Heijnen, Stephan J.P. Hiel, Lotte L. Hoeijmakers, Liselotte Hol, Markus W. Hollmann, Marga E. Hoogendoorn, Janneke Horn, Robrecht van der Horst, Evy L.K. Ie, Dimitri P. Ivanov, Nicole Juffermans, Eline Kho, Eline S. de Klerk, Ankie W.M.M. Koopman-van Gemert, Matty Koopmans, Songul Kucukcelebi, Michael A. Kuiper, Dylan W. de Lange, Niels van Mourik, Sunny G.L.H. Nijbroek, Marisa Onrust, Evelien A.N. Oostdijk, Frederique Paulus, Charlotte J. Pennartz, Janesh Pillay, Luigi Pisani, Ilse M. Purmer, Thijs C.D. Rettig, Jan-Paul Roozeman, Michiel T.U. Schuijt, Marcus J. Schultz, Ary Serpa, Mengalvio E. Sleeswijk, Marry R. Smit, Peter E. Spronk, Willemke Stilma, Aart C. Strang, Anissa M. Tsonas, Pieter R. Tuinman, Christel M.A. Valk, Felicia L. Veen-Schra, Lars I. Veldhuis, Patricia van Velzen, Fleur L.I.M. van der Ven, Ward H. van der Ven, Alexander P.J. Vlaar, Peter van Vliet, Peter H.J. van der Voort, Louis van Welie, Henrico J.F.T. Wesselink, Hermien H. van der Wier-Lubbers, Bas van Wijk, Tineke Winters, Wing Yi Wong, Arthur R.H. van Zanten, EPICCoV, Juliana C. Ferreira, Yeh-Li Ho, Bruno A.M.P. Besen, Luiz M.S. Malbuisson, Leandro U. Taniguchi, IV Pedro Mendes, Eduardo L.V. Costa, Marcelo Park, Renato Daltro-Oliveira, Roberta M.L. Roepke, João M. Silva, Maria José C. Carmona, Carlos Roberto Ribeiro Carvalho, Adriana Hirota, Alberto Kendy Kanasiro, Alessandra Crescenzi, Amanda Coelho Fernandes, Anna Miethke-Morais, Arthur Petrillo Bellintani, Artur Ribeiro Canasiro, Bárbara Vieira Carneiro, Beatriz Keiko Zanbon, Bernardo Pinheiro De Senna Nogueira Batista, Bianca Ruiz Nicolao, Bruno Adler Maccagnan Pinheiro Besen, Bruno Biselli, Bruno Rocha De Macedo, Caio Machado Gomes De Toledo, Carlos Eduardo Pompilio, Carlos Roberto Ribeiro De Carvalho, Caroline Gomes Mol, Cassio Stipanich, Caue Gasparotto Bueno, Cibele Garzillo, Clarice Tanaka, Daniel Neves Forte, Daniel Joelsons, Daniele Robira, Eduardo Leite Vieira Costa, Elson Mendes Da Silva, Fabiane Aliotti Regalio, Gabriela Cardoso Segura, Gustavo Brasil Marcelino, Giulia Sefrin Louro, Yeh-Li Ho, Isabela Argollo Ferreira, Jeison de Oliveira Gois, Joao Manoel Da Silva, Jose Otto Reusing, Julia Fray Ribeiro, Juliana Carvalho Ferreira, Karine Vusberg Galleti, Katia Regina Silva, Larissa Padrao Isensee, Larissa dos Santos Oliveira, Leandro Utino Taniguchi, Leila Suemi Letaif, Lígia Trombetta Lima, Lucas Yongsoo Park, Lucas Chaves, Luciana Cassimiro Nobrega, Luciana Haddad, Ludhmila Hajjar, Luiz Marcelo Malbouisson, Manuela Cristina Adsuara Pandolfi, Marcelo Park, Maria José Carvalho Carmona, Maria Castilho Prandini H De Andrade, Mariana Moreira Santos, Matheus Pereira Bateloche, Mayra Akimi Suiama, Mayron Faria de Oliveira, Mayson Laercio Sousa, Michelle Louvaes, Natassja Huemer, Pedro Mendes, Paulo Ricardo Gessolo Lins, Pedro Gaspar Dos Santos, Pedro Ferreira Paiva Moreira, Renata Mello Guazzelli, Renato Batista Dos Reis, Renato Daltro De Oliveira, Roberta Muriel Longo Roepke, Rodolpho Augusto De Moura Pedro, Rodrigo Kondo, Samia Zahi Rached, Sergio Roberto Silveira Da Fonseca, Thais Sousa Borges, Thalissa Ferreira, Vilson Cobello, Vivian Vieira Tenório Sales, Willaby Serafim Cassa Ferreira, CIBERESUCICOVID, Rafael Mañez, Felipe Rodríguez de Castro, María Mora Aznar, Mateu Torres, María Martinez, Cynthia Alegre, Sofía Contreras, Javier Trujillano, Montse Vallverdú, Miguel León, Mariona Badía, Begoña Balsera, Lluís Servià, Judit Vilanova, Silvia Rodríguez, Neus Montserrat, Silvia Iglesias, Javier Prados, Sula Carvalho, Mar Miralbés, Josman Monclou, Gabriel Jiménez, Jordi Codina, Estela Val, Pablo Pagliarani, Jorge Rubio, Dulce Morales, Andrés Pujol, Àngels Furro, Beatriz García, Gerard Torres, Javier Vengoechea, David de Gozalo Calvo, Jessica González, Silvia Gomez, Lorena Forcelledo Espina, Emilio García Prieto, Paula Martín Vicente, Cecilia del Busto Martínez, María Aguilar Cabello, Carmen Eulalia Martínez Fernández, María Luisa Blasco Cortés, Ainhoa Serrano Lázaro, Mar Juan Díaz, María Teresa Bouza Vieiro, Inés Esmorís Arijón, David Campi Hermoso, Rafaela Nogueras Salinas., Teresa Farre Monjo., Ramon Nogue Bou., Gregorio Marco Naya., Núria Ramon Coll, Juan Carlos Montejo-González, Gloria Renedo Sanchez-Giron, Juan Bustamante-Munguira, Ramon Cicuendez Avila, Nuria Mamolar Herrera, Alexander Agrifoglio, Lucia Cachafeiro, Emilio Maseda, Albert Figueras, Maria Teresa Janer, Laura Soliva, Marta Ocón, Luisa Clar, J Ignacio Ayestarán, Sandra Campos Fernández, Eva Forcadell-Ferreres, Immaculada Salvador-Adell, Neus Bofill, Berta Adell-Serrano, Josep Pedregosa Díaz, Núria Casacuberta-Barberà, Luis Urrelo-Cerrón, Àngels Piñol-Tena, Ferran Roche-Campo, Pablo Ryan Murúa, Covadonga Rodríguez Ruíz, Laura Carrión García, Juan I Lazo Álvarez, Desire Macias Guerrero, Daniel Tognetti, Carlos García Redruello, David Mosquera Rodríguez, Eva María Menor Fernández, Sabela Vara Adrio, Vanesa Gómez Casal, Marta Segura Pensado, María Digna Rivas Vilas, Amaia García Sagastume, Raul de Pablo Sánchez, David Pestaña Laguna, Tommaso Bardi, Carmen Gómez Gonzalez, Maria Luisa Gascón Castillo, José Garnacho-Montero, Joan Ramon Masclans, Ana Salazar Degracia, Judit Bigas, Rosana Muñoz-Bermúdez, Clara Vilà-Vilardel, Francisco Parrilla, Irene Dot, Ana Zapatero, Yolanda Díaz, María Pilar Gracia, Purificación Pérez, Andrea Castellví, Cristina Climent, Lidia Serra, Laura Barbena, Iosune Cano, Alba Herraiz, Pilar Marcos, Laura Rodríguez, Maria Teresa Sariñena, Ana Sánchez, Juan Fernando Masa Jimenez, Gemma Gomà, Mercedes Ibarz, Diego De Mendoza, Enric Barbeta, Victoria Alcaraz-Serrano, Joan Ramon Badia, Manuel Castella, Leticia Bueno, Laia Fernandez Barat, Catia Cillóniz, Pamela Conde, Javier Fernández, Albert Gabarrus, Karsa Kiarostami, Alexandre López-Gavín, Cecilia L Mantellini, Carla Speziale, Nil Vázquez, Hua Yang, Minlan Yang, Carlos Ferrando, Pedro Castro, Marta Arrieta, Jose Maria Nicolas, Rut Andrea, Marta Barroso, Sergio Álvarez, Dario Garcia-Gasulla, Adrián Tormos, Cesar Aldecoa, Rubén Herrán-Monge, José Ángel Berezo García, Pedro Enríquez Giraudo, Pablo Cardinal Fernández, Alberto Rubio López, Orville Báez Pravia, Leire Pérez Bastida, Antonjo Alvarez Ruiz, Anna Parera Pous, Ana López Lago, Eva Saborido Paz, Patricia Barral Segade, Manuel Valledor Mendez, Luciano Aguilera, Esther López-Ramos, Ángela Leonor Ruiz-García, Belén Beteré, Rafael Blancas, Cristina Dólera, Gloria Perez Planelles, Enrique Marmol Peis, Maria Dolores Martinez Juan, Miriam Ruiz Miralles, Eva Perez Rubio, Maria Van der Hofstadt Martin-Montalvo, Tatiana Villada Warrington, Sara Guadalupe Moreno Cano, Federico Gordo, Basilisa Martinez Palacios, Maria Teresa, Sergio Ossa, Ana Ortega, Miguel Sanchez, Bitor Santacoloma, SATI–COVID-19, Elisa Estenssoro, Arnaldo Dubin, Cecilia Inés Loudet, Fernando Ríos, Vanina Siham Kanoore Edul, Gustavo Plotnikow, Rosa Reina Macarena Andrian, Julián Ivacachi, Ignacio Romero, Carla Garay, Damián Piezny, Judith Sagardía, Marco Bezzi, Silvia Borello, Verónica Mandich, Daniel Chiacchiara, Carla Groer, Constanza García Almirón, Ana Kovac, Sebastián Torres, Cristian Cesio, Cristina Orlandi, Rosana Hernández, Paolo Nahuel Rubatto Birri, Matías Mugno, Florencia Valenti, Raúl Alejandro Gómez, Eleonora Cunto, Viviana Chediack, María Gabriela Sáenz, Cecilia Marchena, Norberto Tiribelli, María Guayma, Vanina Aphalo, Daniela Vazquez, Yasmin Saad, Diego Sanchez, Federico Iglesias, Pablo Casteluccio, Bernardo Lattanzio, Sebastián Eiguren, Diego Noval, Sebastián Fredes, Gabriela Izzo, Horacio Cabrera, Mario Pozo, Santiago Sac, Nicolás Tornatore, Julia Sakugawa, Celeste Villafañe, Antonio Di Sibio, Patricio Maskin, Pablo Rodríguez, Nicolás Nihany, Mariela Mogadouro, Fernando Pálizas, Emiliano Cornú, Mariano Esperatti, Juan Manuel Pintos, Gustavo Badariotti, Gonzalo Echevarría, Ana María Mazzola, Cecilia Giuggia, Nahuel Dargains, Alejandra Turano, Florencia Pugliese, Marcos Zec Baskarad, Mariana Chamadoira, Juan Carlos Medina, Marina Búsico, Fernando Villarejo, Hugo Collazos, Tania Huanca, Juan Carlos Pendino, Lionel Talamonti, Fernando Skrzypiec, Claudia Tascón, Gabriela Genovese, Hugo Alul, Agustina Zavattieri, Ana Julieta Herrera, Norma Rosales, María Gabriela Quintana, Alejandro Risso Vazquez, Martín Lugaro, Eduardo Díaz Rousseaux, Marcelo Falcone, Fernando Kurban, Matías Cini, Graciela Zakalik, Carlos Pellegrini, Gabriela Fernández, Juan Pablo Sottile, Sol Barrios, Orlando Hamada, Verónica Mendiluce, Darío Villalba, Florencia Sacco, Vito Mezzina, Carlos Servin, Mónica Quinteros, Hernán Nuñez, María Luz Campassi, David Banegas, Carina Balasini, Victoria Leiva, Franco Maicol, Gustavo Domeniconi, Verónica Vilaseca, Alejandra Barrientos, Florencia Larocca, Liliana Kumar, Rosa Luna, Martín Deheza Lonardi, Agustina Oholeguy, Joaquín Carnero Echegaray, Carla Marazzi, Plácido Helca Regis, Federico Rópolo, Adrián Bobadilla, Vivian Thomas, Nydia Funes Nelson, Cintia Villavicencio, Pedro Machare, Norma Aramayo, Cecilia González, Mariano Ferriccioni, Judith Bergesio

**Affiliations:** 1 Amstrdam University Medical Center Department of Intensive Care Amsterdam Netherlands Department of Intensive Care, Amstrdam University Medical Center, Location AMC - Amsterdam, Netherlands.; 2 Hospital Israelita Albert Einstein Department of Intensive Care São Paulo SP Brazil Department of Intensive Care, Hospital Israelita Albert Einstein – São Paulo (SP), Brazil.; 3 University or Trento Centre for Medical Sciences Trento Italy Centre for Medical Sciences, University or Trento - Trento, Trentino-Alto Adige, Italy.; 4 Hospital Interzonal de Agudos General San Martin La Plata Department of Intensive Care Buenos Aires Argentina Department of Intensive Care, Hospital Interzonal de Agudos General San Martin La Plata - Buenos Aires, Argentina.; 5 University of Toronto Institute of Health Policy, Management and Evaluation Interdepartmental Division of Critical Care Medicine Ontario Canada Interdepartmental Division of Critical Care Medicine, and Institute of Health Policy, Management and Evaluation, University of Toronto - Ontario, Canada.; 6 Universidade de São Paulo Faculdade de Medicina Hospital das Clínicas São Paulo SP Brazil Department of Pulmonology, Hospital das Clínicas, Faculdade de Medicina, Universidade de São Paulo - São Paulo (SP), Brazil.; 7 Saolta Hospital Group Galway University Hospital Department of Anaesthesiology and Intensive Care Galway Ireland Department of Anaesthesiology and Intensive Care, Galway University Hospital, Saolta Hospital Group - Galway, Ireland.; 8 Multidisciplinary Intensive Care Research Organization, St James’ Hospital Department of Intensive Care Dublin Ireland Department of Intensive Care, Multidisciplinary Intensive Care Research Organization, St James’ Hospital - Dublin, Ireland.; 9 Hospital Clínic de Barcelona Institut d’Investigacions Biomèdiques August Pi i Sunyer Department of Pulmonology Barcelona Spain Department of Pulmonology; Institut d’Investigacions Biomèdiques August Pi i Sunyer, Hospital Clínic de Barcelona - Barcelona, Spain.; 10 Université Paris Equipe d’Epidémiologie Respiratoire Integrative Paris France Equipe d’Epidémiologie Respiratoire Integrative, Université Paris–Saclay - Paris, France.; 11 Institute of Health Carlos III Centro de Investigación Biomédica en Red en Enfermedades Respiratorias Madrid Spain Centro de Investigación Biomédica en Red en Enfermedades Respiratorias, Institute of Health Carlos III - Madrid, Spain.; 12 Amstrdam University Medical Center Laboratory of Experimental Intensive Care & Anaesthesiology Amsterdam Netherlands Laboratory of Experimental Intensive Care & Anaesthesiology, Amstrdam University Medical Center, Location AMC - Amsterdam, Netherlands.

**Keywords:** Intensive care, Critical care, Respiration, artificial, Pandemics, Coronavirus disease, COVID-19, Acute respiratory distress syndrome, ARDS, Mortality, Survival

## Abstract

**Background:**

Mortality rates among patients with acute respiratory distress syndrome (ARDS) due to COVID-19 vary widely, and there is a lack of studies comparing mortality patterns in COVID-ARDS to ARDS caused by other respiratory infections (non-COVID-ARDS).

**Methods:**

A post hoc analysis was performed on a database comprising individual patient data from four observational studies on COVID-19 and two pre-pandemic studies; the analysis was limited to patients with ARDS receiving mechanical ventilation. Propensity score matching was used to control for factors known to be associated with mortality and for geographic region.

**Results:**

The analysis included 8,117 patients: 6,702 with COVID-ARDS and 1,415 with non-COVID-ARDS. Lung-protective ventilation, including low tidal volume and high positive end-expiratory pressure, was more often applied in the COVID-ARDS group. In unmatched analysis, mortality at day 28 and day 60 was 37.0 *versus* 33.5% (p = 0.012) and 44.2 *versus* 36.4% (p = 0.002) in COVID-ARDS *versus* non-COVID-ARDS, respectively. In a matched analysis, including 4,580 patients with COVID-ARDS and 1,274 patients with non-COVID-ARDS, mortality at days 28 and 60 was 35.7% *versus* 33.5% (p = 0.149) and 42.5% *versus* 36.5% (p = 0.002), respectively. In a matched analysis controlling for geographic region, mortality at days 28 and 60 did not differ between COVID-ARDS and non-COVID-ARDS.

**Conclusion:**

In an unmatched analysis, patients with COVID-ARDS had higher mortality at day 28 and day 60 than patients with non-COVID-ARDS; after adjusting for risk factors, only 60-day mortality remained higher, and after adjusting for geographic region, mortality rates were similar.

## INTRODUCTION

The outbreak of the highly contagious severe acute respiratory syndrome coronavirus type 2 impacted healthcare systems worldwide. Considerable numbers of patients who developed coronavirus disease 2019 (COVID-19) required hospital admission for supplementary oxygen, and a significant proportion of patients needed care in an intensive care unit (ICU) for escalation of respiratory support.^(1)^ The initial mortality rates of patients who developed severe hypoxemic respiratory failure in China, where the pandemic originated, were extremely high.^(2)^ Subsequently, lower rates were reported, although significant differences in mortality rates persisted.^(3–7)^

During the initial stages of the pandemic, uncertainty surrounded COVID-acute respiratory distress syndrome (ARDS). It shares many similarities with ARDS due to other respiratory infections (non-COVID-ARDS)^(1,8–13)^ but also has specific differences.^(11,14–16)^ The evolving nature of understanding the novel disease might have led to variations in clinical approaches, at least initially, affecting patient care and, consequently, influencing outcomes. The overwhelming number of patients needing ICU care during the pandemic may have further strained healthcare resources, potentially contributing to differences in care delivery and subsequent outcomes for COVID-ARDS patients compared to non-COVID-ARDS patients.

We assessed a database comprising individual patient data from four observational studies on acute hypoxemic failure related to COVID-19 and from two studies conducted before the pandemic. We compared outcomes in patients with COVID-ARDS with those in patients with non-COVID-ARDS. We used propensity score matching to control for factors known to be associated with mortality and for geographic region. We hypothesize that mortality at days 28 and 60 will be similar in COVID-ARDS and non-COVID-ARDS patients.

## METHODS

### Study design

Post hoc analysis of a database that pooled the individual data of patients included in Epidemiology of Respiratory Insufficiency in Critical Care (ERICC),^(17)^ Large Observational study to UNderstand the Global impact of Severe Acute respiratory FailurE (LUNG SAFE),^(18)^ Practice of Ventilation in COVID-19 patients (PRoVENT-COVID),^(19)^ EPIdemiology of Critical COVID-19 patients in the ICU (EPICCoV),^(20)^ *Centro de Investigación Biomédica en Red Enfermedades Respiratorias* COVID-19 (CIBERESUCICOVID),^(21)^ and Clinical Characteristics and Outcomes of Patients with COVID-19 on Mechanical Ventilation in Argentina (SATI-COVID-19).^(22)^ The individual study protocols were all approved by an Institutional Review Board, and for all studies, the need for individual patient informed consent was waived due to the observational nature. Other details regarding ethics can be found in the original publications.

We invited the corresponding authors of these studies to participate in the pooling of the individual patient data. After their consent, we received the case report forms and data dictionaries. The patient data received were harmonized and subsequently merged. Creating the pooled database did not require additional ethical approval. The pooled database is registered at clinicaltrials.gov (identifier NCT05650957).

### Inclusion and exclusion criteria

The inclusion and exclusion criteria used in the original studies are available in the original publications. For the current analysis, patients were eligible if: aged > 17 years; receiving ventilation for acute hypoxemic failure; due to a respiratory infection. As we wanted to compare outcome patterns in COVID-ARDS *versus* non-COVID-ARDS, we excluded patients without ARDS according to the Berlin definition,^(23)^ patients in whom COVID-19 was not confirmed, and patients with another cause of ARDS. We also excluded those with an incomplete follow-up.

### Available data in the pooled database

The pooled database contains the following data: patient demographic and baseline characteristics, including age, sex, weight and height, comorbidities, and the Sequential Organ Failure Assessment (SOFA) score at baseline; ventilation characteristics, including ventilation mode; tidal volume (V_T_); peak pressure (Ppeak) in volume-controlled ventilation, plateau pressure (Pplat) in pressure-controlled ventilation, positive end-expiratory pressure (PEEP); fraction of inspired oxygen (FiO_2_), and respiratory rate (RR); data on gas exchange, including full arterial blood gas analyses results; provided supportive care, including prone positioning, continuous infusion of neuromuscular blocking agents, and extracorporeal membrane oxygenation (ECMO); and outcomes, including last day of ventilation, last day in ICU and hospital, ICU and in-hospital mortality, and life status after hospital discharge up to day 60 or 90, depending on the original study.

### Calculations

Tidal volume is expressed per predicted body weight (PBW), a calculation based on sex and height.^(24)^ Static and dynamic lung compliance were calculated using standard equations.^(25)^ Driving pressure (ΔP) was calculated only at timepoints with evidence of absence of spontaneous breathing, by subtracting PEEP from Pplat during volume-controlled ventilation, or from Pmax during pressure-controlled ventilation.^(26,27)^ Respiratory system compliance (C_RS_) was calculated by dividing V_T_ by ΔP. Mechanical power (MP) was calculated using the following power equation:^(28)^ MP (J/min) = 0.098 * V_T_ * RR * (Ppeak-0.5 * ΔP).^(28)^ The following modified power equation was used if Ppeak was not available: 0.098 * V_T_ * RR * (Pplat-0.5 * ΔP).^(27)^

The number of days free from ventilation and alive at day 28 (ventilation-free days [VFD]-28) and day 60 (VFD-60) was calculated as described before.^(29)^ Herein, we followed the method of Schoenfeld et al.,^(30)^ and VFD was assigned zero not only in patients alive and mechanically ventilated at day 28 or day 60 respectively, but also in patients who had died by day 28 or day 60 respectively. In these variables, only the first intubation was used, as data on reintubation were often missing.

### Endpoints

The co-primary endpoints of this analysis were 28- and 60-day mortality. Secondary endpoints included ICU and hospital mortality, ventilation duration in survivors, VFD-28 and VFD-60, and ICU and hospital length of stay in survivors.

### Power calculation

We did not perform a power calculation; the number of patients in the database served as the sample size.

### Statistical analysis

Continuous variables are reported as medians with interquartile ranges, categorical variables as numbers and relative proportions, where appropriate.

Demographic, baseline, and ventilation characteristics are reported and compared between survivors and non-survivors through day 60, using t-tests for continuous variables and Chi-squared tests for categorical variables. Ventilation parameters are visualized in cumulative distribution plots, and data regarding adjunctive respiratory support are presented in tables.

Hazard ratios (HRs) for the outcomes of interest were compared between COVID-ARDS and non-COVID-ARDS using a shared frailty model with the patient as the frailty factor. Survival for both groups is visualized in Kaplan-Meier curves.

A competing-risks model was developed to estimate the effects of COVID-ARDS and non-COVID-ARDS on outcomes, with death and extubation as competing events. For this model, the Fine-Gray proportional hazards model for subdistribution^(31)^ was used, and cumulative incidence functions were calculated and visualized in plots.

For propensity score matching, COVID-ARDS patients were matched to non-COVID-ARDS patients’ infections in a 4:1 ratio, with a maximum caliper of 0.02 using covariate propensity score balancing.^(32)^ The nearest neighbor matching strategy was used for matching. The following variables were used for propensity matching: age, gender, body mass index (BMI), partial arterial oxygen level (PaO_2_)/FiO_2_, plasma creatinine, fluid balances, a history of hypertension, heart failure, diabetes, chronic obstructive pulmonary disease, or malignancy, arterial pH, mean arterial pressure and heart rate. These baseline variables were selected because of clinical relevance. In a second step, we also matched COVID-ARDS patients with non-COVID-ARDS patients across two geographic regions: Europe and South America.

All analyses were conducted in R (v.4.2.1) (Vienna, Austria), and a p value < 0.05 was considered statistically significant. No correction for multiple testing was performed, as all analyses should be seen as exploratory.

## RESULTS

### Patients

**Figure 1:**
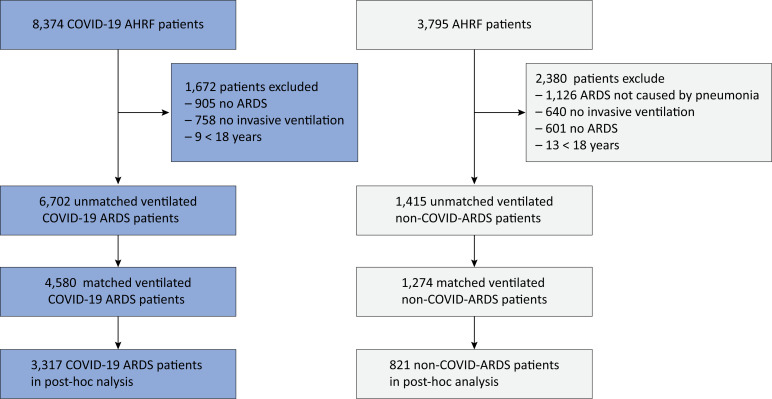
Patient flow.

From the pooled database, we included 6,702 COVID-ARDS patients and 1,415 non-COVID-ARDS patients (Figure 1). The main reasons for exclusion were not having received invasive ventilation, not having ARDS according to the Berlin definition, or not having ARDS that was caused by a respiratory infection. The matched cohort included 4,580 COVID-ARDS patients and 1,274 non-COVID-ARDS patients. Survivors were younger, had comorbidities less often, and were classified as having mild ARDS more often, in the unmatched (Table 1) and in the matched cohort (Table 1S - Supplementary Material).

AHRF - acute hypoxemic respiratory failure; ARDS - acute respiratory distress syndrome.

**Table 1:** Patient demographics and baseline characteristics in survivors *versus* nonsurvivors

		Survivors			Nonsurvivors		
		COVID-ARDS (n = 3,738)	Non-COVID-ARDS (n = 900)	p value	COVID-ARDS (n = 2,964)	Non-COVID-ARDS (n= 515)	p value
Age (years)		61.0 [52.0 - 69.0]	61.0 [48.0 - 72.0]	0.358	67.0 [59.0 - 73.0]	67.0 [56.0 - 76.0]	0.192
Sex, male		2,535 (67.9)	542 (60.2)	0.009	2,120 (71.6)	317 (61.6)	0.012
Height (cm)		170.0 [164.0 - 176.0]	169.0 [160.0 - 175.0]	0.004	170.0 [162.0 - 175.0]	168.0 [160.0 - 174.0]	0.018
Weight (kg)		84.0 [75.0 - 95.0]	75.0 [65.0 - 89.0]	< 0.001	80.0 [72.0 - 92.0]	70.0 [60.0 - 85.0]	< 0.001
BMI (kg/m^2^)		28.7 [25.7 - 32.5]	26.1 [22.9 - 30.8]	< 0.001	28.3 [25.5 - 32.1]	24.9 [22.0 - 29.3]	< 0.001
SOFA score		6.0 [4.0 - 8.0]	9.0 [7.0 - 12.0]	< 0.001	7.0 [4.0 - 9.0]	11.0 [8.0 - 14.0]	< 0.001
Comorbidities							
	Cardiac failure	271 (7.3)	83 (9.2)	0.045	362 (12.2)	60 (11.7)	0.718
	COPD	310 (8.3)	207 (23.0)	< 0.001	378 (12.8)	116 (22.5)	< 0.001
	Diabetes	902 (24.1)	194 (21.6)	0.103	1004 (33.9)	128 (24.9)	0.037
	CKD	157 (4.2)	76 (8.4)	0.002	245 (8.3)	64 (12.4)	0.002
	Liver failure		21 (2.3)	0.058	68 (2.3)	32 (6.2)	0.008
	Cancer	98 (2.6)	53 (5.9)	0.006	155 (5.2)	66 (12.8)	< 0.001
ARDS severity				0.008			< 0.001
	Mild	791 (21.2)	257 (28.6)		497 (16.8)	123 (23.9)	
	Moderate	1981 (53.0)	433 (48.1)		1530 (51.6)	244 (47.4)	
	Severe	966 (25.8)	210 (23.3)		937 (31.6)	148 (28.7)	

ARDS - acute respiratory distress syndrome; BMI - body mass index; SOFA - Sequential Organ Failure Assessment; COPD - chronic obstructive pulmonary disease; CKD - chronic kidney disease. Data expressed as median [interquartile range] or n (%).

### Ventilatory support characteristics

**Figure 2:**
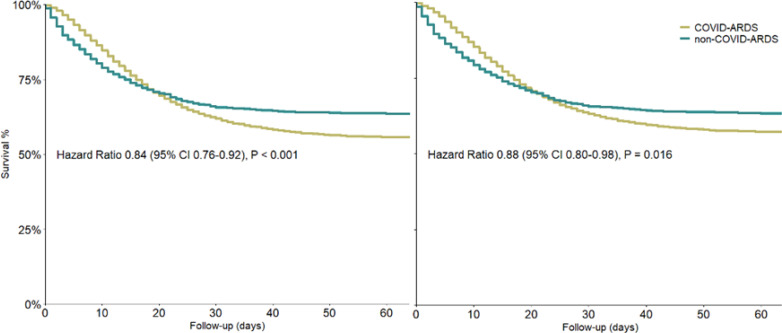
Kaplan-Meier curves for mortality in the unmatched and the matched cohorts.

In the unmatched cohort, COVID-ARDS patients received ventilation with lower median V_T_ and lower median ΔP, but higher median RR, median PEEP, median Ppeak, median C_RS_, and higher median MP compared to non-COVID-ARDS patients (Table 2 and Figure 1S and 2S [Supplementary Material]). Adjunctive therapies were more often used in COVID-ARDS patients. In the matched cohort, similar differences were seen.

**Table 2:** Ventilation characteristics, adjunctive therapies and arterial blood gas analysis results in survivors *versus* nonsurvivors

			Survivors			Nonsurvivors		
			COVID-ARDS (n = 3,738)	Non-COVID-ARDS (n = 900)	p value	COVID-ARDS (n = 2,964)	Non-COVID-ARDS (n = 515)	p value
Ventilation characteristics								
	Mode of ventilation				< 0.001			< 0.001
		Volume-controlled	2,555 (68.6)	327 (36.3)		2,134 (72.4)	194 (37.7)	
		Pressure-controlled	830 (22.3)	309 (34.3)		573 (19.5)	184 (35.7)	
		Pressure-support	189 (5.1)	95 (10.6)		147 (5.0)	48 (9.3)	
		Other	150 (4.0)	169 (18.8)		92 (3.1)	89 (17.3)	
	V_T_ (mL/kg PBW)		6.7 [6.0 - 7.4]	7.3 [6.3 - 8.4]	< 0.001	6.5 [6.0 - 7.4]	7.3 [6.4 - 8.5]	< 0.001
		≤ 6	773 (23.5)	135 (16.4)		691 (26.6)	76 (16.5)	
		6 - 8	2,042 (62.1)	418 (51.0)		1,559 (60.0)	227 (49.2)	
		8-10	398 (12.1)	201 (24.5)		300 (11.5)	123 (26.7)	
		> 10	77 (2.3)	67 (8.2)		48 (1.8)	35 (7.6)	
	PEEP (cmH_2_O)		12.0 [10.0 - 14.0]	8.0 [6.0 - 10.0]	< 0.001	12.0 [10.0 - 14.0]	8.0 [6.0 -10.0]	< 0.001
		≤ 8	472 (12.6)	494 (54.9)		540 (18.2)	302 (58.6)	
		8 - 12	1,902 (50.9)	295 (32.8)		1,486 (50.1)	152 (29.5)	
		12 - 16	1,257 (33.6)	89 (9.9)		844 (28.5)	51 (10.0)	
		> 16	107 (2.9)	22 (2.4)		94 (3.2)	10 (2.0)	
	Pmax (cmH_2_O)		31.0 [28.0 - 35.0]	29.0 [24.0 - 34.0]	< 0.001	32.0 [28.0 - 36.0]	30.0 [25.0 - 35.0]	0.059
	ΔP (cmH_2_O)		12.0 [10.0 - 15.0]	16.0 [12.0 - 20.0]	< 0.001	13.0 [10.0 - 16.0]	17.0 [13.0 - 21.0]	< 0.001
	Crs (mL/cmH_2_O)		34.6 [27.9 - 43.3]	28.6 [22.5 - 38.7]	0.010	32.2 [25.1 - 40.9]	26.4 [20.5 - 37.1]	0.024
	MP (J/minute)		23.8 [18.6 - 28.9]	20.8 [16.1 - 28.6]	0.433	23.6 [18.8 - 29.9]	22.1 [16.1 - 29.0]	0.218
	FiO_2_		0.60 [0.50 - 0.89]	0.60 [0.45 - 0.90]	0.361	0.70 [0.50 - 1.00]	0.65 [0.50 - 1.00]	0.574
	Total RR (breaths/minute)		22.0 [20.0 - 25.0]	20.0 [16.0 - 25.0]	< 0.001	22.0 [20.0 - 26.0]	20.0 [16.0 - 25.0]	0.017
Adjunctive therapies								
	Prone positioning		2480 (66.7)	91 (10.1)	< 0.001	2,135 (72.5)	53 (10.3)	< 0.001
	Recruitment maneuvers		1136 (39.0)	179 (19.9)	< 0.001	788 (40.1)	134 (26.0)	< 0.001
	ECMO		78 (2.3)	44 (4.9)	0.033	64 (2.8)	12 (2.3)	0.538
	Tracheostomy		1,402 (37.7)	157 (17.4)	< 0.001	631 (21.4)	57 (11.1)	0.003
	Neuromuscular blocking agents		2,294 (73.2)	204 (22.7)	< 0.001	1,544 (74.9)	146 (28.4)	< 0.001
	Continuous sedation		982 (98.8)	760 (84.4)	< 0.001	803 (97.3)	428 (83.1)	< 0.001
	Vasopressor use		2,610 (83.4)	598 (66.4)	< 0.001	1,830 (88.7)	427 (83.1)	< 0.001
Arterial blood gas								
	pH		7.37 [7.31 - 7.42]	7.35 [7.28 - 7.42]	0.003	7.32 [7.25 - 7.39]	7.32 [7.21 - 7.40]	0.008
	PaO_2_ (mmHg)		81.0 [68.0 - 99.0]	84.0 [69.0 - 105.8]	< 0.001	80.0 [67.0 - 97.5]	83.0 [66.6 - 105.7]	0.012
	PaCO_2_ (mmHg*)		43.5 [37.9 - 50.3]	44.6 [37.5 - 53.9]	0.003	45.8 [39.0 - 54.2]	43.8 [36.0 - 54.0]	0.351
	PaO_2_/FiO_2_		140.0 [98.2 - 190.0]	149.6 [102.5 - 210.0]	0.019	130.0 [88.9 - 176.4]	140.0 [92.0 - 198.0]	0.004

ARDS - acute respiratory distress syndrome; V_T_ - tidal volume; PBW - predicted bodyweight; PEEP - positive end–expiratory pressure; Pmax - maximum airway pressure; ΔP - driving pressure; Crs - respiratory system compliance; MP - mechanical power; FiO_2_ - fraction of inspired oxygen; RR -respiratory rate; ECMO - extracorporeal membrane oxygenation; PaO_2_ - partial pressure of arterial oxygen; PaCO_2_ - partial pressure of arterial carbon dioxide. Data expressed as n (%) or median [interquartile range].

### Outcomes of the unmatched analysis

**Figure 3:**
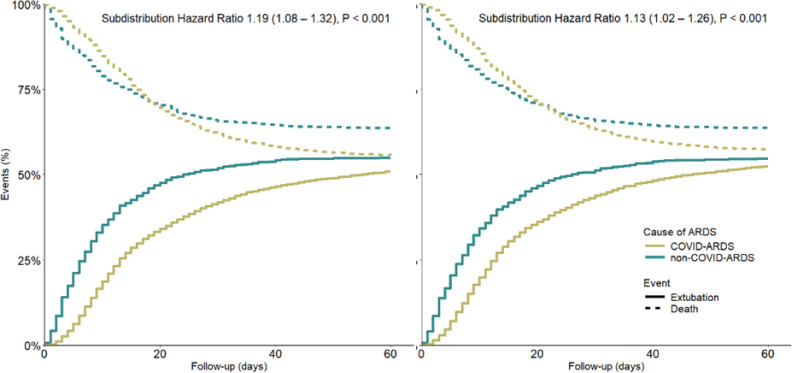
Competing Risk Model in the unmatched and the matched cohorts.

In the unmatched cohort, 2,477 (37.0%) COVID-ARDS patients had died at day 28 *versus* 474 (33.5%) non-COVID-ARDS patients. At day 60, mortality rates were 2,964 (44.2%) patients *versus* 515 (36.4%) patients, respectively (HR 0.84 [0.76 - 0.92]; p < 0.001) (Table 3 and Figure 2). COVID-ARDS patients had lower VFD-28 and VFD-60 scores, a longer duration of ventilation, and a longer ICU length of stay (Table 3 and Figure 3). Length of hospital stay did not differ between COVID-ARDS patients and non-COVID-ARDS patients.

**Table 3:** Outcomes in the unmatched and the matched cohorts

		Unmatched cohort			Matched cohort		
		COVID-ARDS (n = 6,702)	Non-COVID-ARDS (n = 1,415)	p value	COVID–ARDS (n = 4,580)	Non-COVID-ARDS (n = 1,274)	p value
Mortality rates							
	28 day	2,477 (37.0)	474 (33.5)	0.012	1,631 (35.7)	427 (33.5)	0.149
	60 day	2,964 (44.2)	515 (36.4)	0.002	1,948 (42.5)	465 (36.5)	0.002
	ICU	2,806 (43.6)	518 (36.6)	0.003	1,844 (41.9)	469 (36.8)	0.001
	Hospital	2,976 (45.1)	525 (37.1)	0.001	1,962 (43.5)	475 (37.3)	0.047
VFD-28 (days)		0.0 [0.0 - 15.0]	4.0 [0.0 - 21.0]	< 0.001	0.0 [0.0 - 15.0]	2.5 [0.0 - 21.0]	< 0.001
VFD-60 (days)		5.0 [0.0 - 47.0]	36.0 [0.0 - 53.0]	< 0.001	13.0 [0.0 - 47.0]	34.0 [0.0 - 53.0]	0.001
Duration of ventilation (days)		14.0 [8.0 - 25.0]	8.0 [3.0 - 15.0]	< 0.001	14.0 [8.0 - 24.0]	8.0 [3.0 -15.0]	< 0.001
Length of stay in ICU		17.0 [10.0 - 29.0]	10.0 [5.0 - 19.0]	< 0.001	17.0 [10.0 - 29.0]	11.0 [5.0 - 19.0]	< 0.001
Length of stay in hospital		25.0 [15.0 - 41.0]	25.0 [14.0 - 41.0]	0.728	25.0 [15.0 - 41.0]	26.0 [15.0 - 43.0]	0.194

ARDS - acute respiratory distress syndrome; ICU - intensive care unit; VFD - ventilator-free days. Data expressed as n (%) or median [interquartile range].

ARDS - acute respiratory distress syndrome; 95%CI - 95% confidence interval.

ARDS - acute respiratory distress syndrome; 95%CI - 95% confidence interval.

### Outcomes of the matched analyses

After propensity score matching, only 60-day mortality was significantly different between COVID-ARDS patients and non-COVID-ARDS patients (Table 3 and Figure 2). After including the geographic regions Europe and South America as covariates in the propensity score matching, the mortality rates at day 28 and 60 were similar between both groups (Table 4). The love plots for the matched analyses are shown in figures 3S and 4S (Supplementary Material).

**Table 4:** Mortality in the unmatched cohort and after post-hoc analysis

		Unmatched cohort			Matched cohort		
		COVID–ARDS n = 6,702)	Non-COVID-ARDS (n = 1,415)	p value	COVID–ARDS (n = 3,317)	Non-COVID-ARDS (n = 821)	p value
Mortality rates							
	28 day	2,477 (37.0)	474 (33.5)	0.012	1,058 (32.0)	278 (33.9)	0.304
	60 day	2,946 (44.2)	515 (36.4)	0.002	1,278 (38.5)	310 (37.8)	0.685

ARDS - acute respiratory distress syndrome. Data expressed as n (%)

## DISCUSSION

The findings of this post hoc analysis of a pooled database of four observational studies in COVID-ARDS patients and two studies originating before the pandemic can be summarized as follows: in unmatched analysis, COVID-ARDS patients exhibited a higher 28- and 60-day mortality compared to non-COVID-ARDS patients; after propensity score matching for risk factors for death only 60-day mortality was different, and after propensity score matching for geographic region mortality rates were similar.

Our analysis has several strengths. We effectively compiled and integrated datasets from two pre-pandemic observational studies and four studies early in the COVID-19 pandemic. The studies reported causes of ARDS, facilitating the selection of patients for the current analysis. The inclusion of global datasets with diverse patient demographics strengthens the generalizability of the findings. The original datasets were notably comprehensive, covering patient demographics, baseline characteristics, ventilator settings, ventilation parameters, and key clinical outcomes. This allowed seamless integration into a single extensive database, supporting intricate statistical analyses due to the substantial patient cohort.

This is one of the first analyses to compare outcomes of COVID-ARDS to non-COVID-ARDS using a sufficiently large database to allow for association analyses and propensity score matching to correct for confounders. In contrast to reports early in the COVID-19 pandemic, suggesting very high mortality rates in COVID-ARDS,^(5,6,10)^ we found a mortality rate that is remarkably similar to that in ARDS that is not related to COVID-19. In fact, our findings confirm the mortality rates in patients with ARDS from before the pandemic^(33–35)^ and those in patients with COVID-ARDS later in the pandemic.^(36–38)^ These outcomes add to the growing body of evidence that differences in pathophysiology and even management between COVID-ARDS and non-COVID-ARDS do not translate into differences in outcomes.^(38–41)^

In line with previous studies,^(1,42)^ our analysis shows that lung protective ventilation, with low volumes and low pressures, was more often applied in COVID-ARDS patients compared to patients with ARDS before the COVID pandemic. Low V_T_ ventilation has been shown to reduce mortality and morbidity in ARDS patients well before the pandemic,^(9,43)^ yet remained underused.^(44)^ In our study, the difference in V_T_ between patients with COVID-ARDS and non-COVID-ARDS is statistically significant, and this may be an explanation for the difference in mortality at day 28. We also show that higher PEEP was used more often in COVID-ARDS patients compared to non-COVID-ARDS patients. This is in line with observations from other studies.^(9,43)^ Despite the adverse effects of high PEEP^(45)^ and the absence of benefit in studies in COVID-ARDS patients.^(46)^ High PEEP could have a reducing effect on early mortality in patients with subsequent improved oxygenation, but might result in barotrauma, especially in patients without improved oxygenation, resulting in a neutral effect on late mortality.^(47)^ This hypothesis might contribute to the difference in early mortality seen in our matched analysis, which disappeared at day 60.

Even though 28-day and 60-day mortality were significantly higher in COVID-ARDS patients compared to non-COVID-ARDS patients in the unmatched analysis, the difference in 28-day mortality disappeared after matching. Since several studies suggest that geographic variation may affect outcomes in ventilated ICU patients,^(48,49)^ we also performed a matched analysis by geographic region. In this analysis, the difference in day 60 mortality disappeared, leaving no differences in mortality rates. This aligns with previous studies reporting significant geographic disparities in COVID-ARDS mortality, driven by differences in socio-economic contexts and vaccination rates.^(50,51)^ These potential influences highlight the importance of further investigation, and future studies should account for the study region and consider expanding the focus to later timepoints when mortality is the endpoint.

Our analysis also shows that COVID-ARDS patients had a longer dependence on invasive mechanical ventilation, had fewer ventilator-free days, and a more extended ICU stay. These results are consistent with previous analysis comparing COVID-ARDS patients to non-COVID-ARDS patients.^(10,52,53)^

Our analysis has limitations. Data originated from observational studies, allowing for associations but not for causality. Moreover, the willingness of certain hospitals to share data in the original studies could have introduced selection bias by favoring the inclusion of units with a strong interest in ARDS. The studies on COVID-ARDS were conducted in the early stages of the pandemic, characterized by strained hospital capacities, at times with limited resources and possibly even inexperienced staff, which influenced patient care and contributed to missing information. Data on reintubation, for example, were often missing, thereby influencing outcomes such as ventilation duration and VFDs. Differences in resource allocation and staff experience could also contribute to disparities in regional outcomes. Furthermore, in non-COVID-ARDS patients, it was not possible to specify if a bacterial, viral, or fungal pathogen caused the respiratory infection. Lastly, significant disparities emerged among the cohorts once regional factors were taken into account.

## CONCLUSION

In an unmatched analysis, patients with COVID-ARDS had higher mortality at day 28 and day 60 than non-COVID-ARDS patients; after adjusting for risk factors, only 60-day mortality remained higher, and after adjusting for geographic region, mortality rates were similar. Further research should prioritize longer-term outcomes and incorporate regional factors to enhance the understanding of global disparities in ARDS management.

## Data Availability

A de-identified dataset will be made available upon request to the corresponding authors one year after publication of this study. The request must include a statistical analysis plan.
