# Supplementary material for: Outcome patterns in COVID-ARDS compared to ARDS caused by other respiratory infections: an individual patient data analysis of six observational studies
Source: Crit Care Sci. 2026 Jan 28;38:e20260182. doi: 10.62675/2965-2774.20260182 (PMC12977218; doi:10.62675/2965-2774.20260182)
Supplement: SUPPLEMENTARY MATERIAL [file 2965-2774-ccsci-38-e20260182-suppl1.pdf]

Critical Care Science
https://doi.org/10.62675/2965-2774.20260182

SUPPLEMENTARY MATERIAL

Outcome patterns in COVID-ARDS compared to ARDS caused by other respiratory infections: an individual patient data analysis of six observational studies

Paula J. van Oosten[†1], Siebe G. Blok[†1], Pien Swart[1], Luciano Cesar Pontes Azevedo[2], Giacomo Bellani[3], Michela Botta[1], Elisa Estenssoro[4], Eddy Fan[5], Juliana Carvalho Ferreira[6], John G. Laffey[7], Ignacio Martin-Loeches[8], Anna Motos[9], Tai Pham[10], Oscar Peñuelas[11], Marcus J. Schultz[12], Ary Serpa Neto[2], Antoni Torres Marti[9], Anissa M. Tsonas[1], Frederique Paulus[1], David Michael Paul van Meenen[1] for the ERICC[a], LUNG SAFE[b], PRoVENT–COVID[c], EPICCoV[d], CIBERESUCICOVID[e] and SATI–COVID-19[f] investigators

Table 1S - Patient demographics and baseline characteristics in survivors *versus* nonsurvivors, in matched patients

	Survivors			Nonsurvivors		
	**COVID-ARDS (n = 2,632)**	**Non-COVID-ARDS (n = 809)**	**p value**	**COVID-ARDS (n = 1,948)**	**Non-COVID-ARDS (n = 465)**	**p value**
Age (years)	61.0 [52.0 - 69.0]	61.0 [48.0 - 73.0]	0.792	67.0 [59.0 - 73.0]	67.0 [56.0 - 76.0]	0.446
Sex, male	1,687 (64.1)	501 (61.9)	0.263	1,317 (67.6)	291 (62.6)	0.039
Height (cm)	170.0 [163.0 - 176.0]	170.0 [162.0 - 175.0]	0.002	170.0 [162.0 - 175.0]	168.0 [160.0 - 174.0]	0.002
Weight (kg)	80.0 [70.0 - 90.0]	75.0 [65.0 - 90.0]	0.007	80.0 [70.0 - 90.0]	70.0 [60.0 - 85.0]	< 0.001
BMI (kg/m²)	27.7 [25.0 - 31.2]	26.2 [23.0 - 30.8]	<0.001	27.6 [24.8 - 31.1]	25.1 [22.0 - 29.4]	< 0.001
SOFA score	6.0 [4.0 - 8.0]	9.0 [7.0 - 12.0]	<0.001	7.0 [4.0 - 9.0]	11.0 [8.0 - 14.0]	< 0.001
Comorbidities						
Cardiac failure	188 (7.1)	78 (9.6)	0.020	250 (12.8)	55 (11.8)	0.558
COPD	285 (10.8)	174 (21.5)	<0.001	329 (16.9)	97 (20.9)	0.044
Diabetes	566 (21.5)	172 (21.3)	0.883	626 (32.1)	116 (25.0)	0.003
CKD	137 (5.2)	67 (8.3)	0.001	209 (10.7)	53 (11.4)	0.677
Liver failure	50 (1.9)	17 (2.1)	0.717	59 (3.0)	24 (5.2)	0.023
Cancer	87 (3.3)	44 (5.4)	0.006	136 (7.0)	57 (12.3)	< 0.001
ARDS severity			0.007			0.088
Mild	604 (22.9)	227 (28.1)		372 (19.1)	106 (22.8)	
Moderate	1,391 (52.8)	386 (47.7)		1,023 (52.5)	220 (47.3)	
Severe	637 (24.2)	196 (24.2)		553 (28.4)	139 (29.9)	

ARDS - acute respiratory distress syndrome; BMI - body mass index; SOFA - Sequential Organ Failure Assessment; COPD - chronic obstructive pulmonary disease; CKD - chronic kidney disease. Data are median [interquartile range] or n (%).

This is an open access article under the CC BY license (https://creativecommons.org/licenses/by/4.0/).

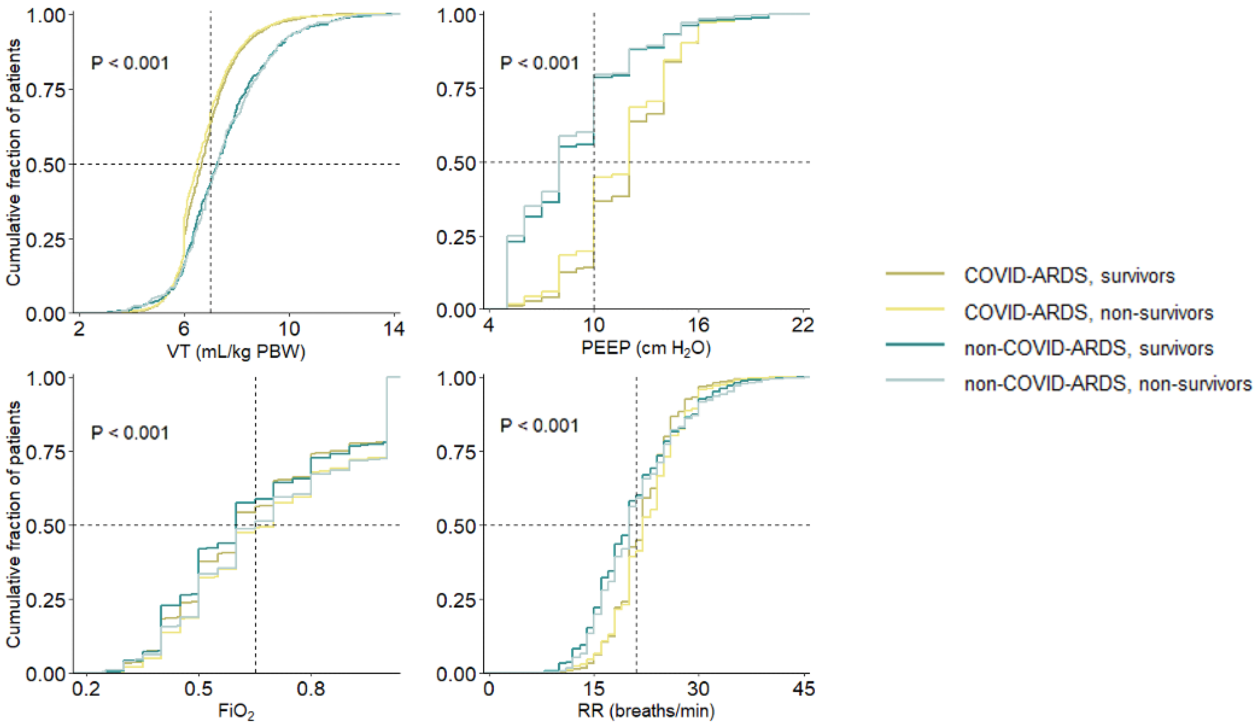

V_T - tidal volume; PBW - predicted bodyweight; PEEP - positive end–expiratory pressure; FiO_2 - fraction of inspired oxygen; RR - respiratory rate; ARDS - acute respiratory distress syndrome.

Figure 1S - Cumulative distribution of ventilator settings.

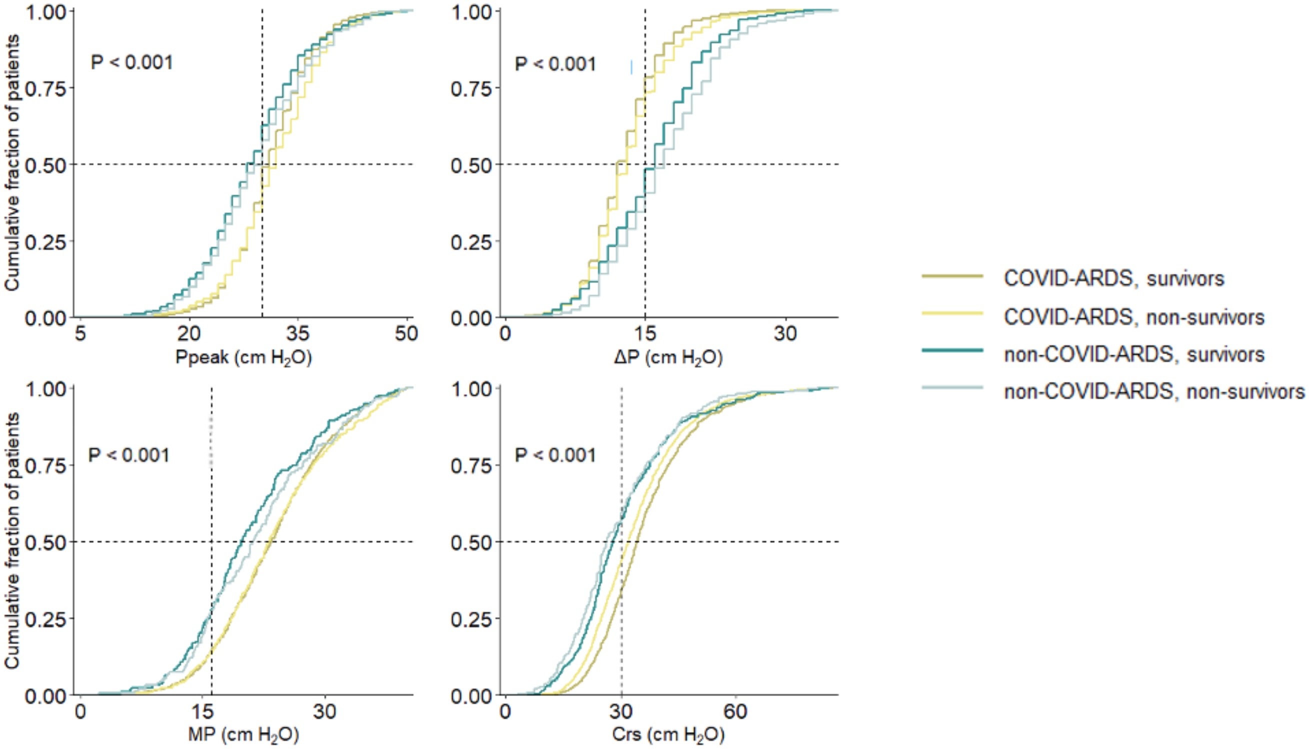

Ppeak - maximum airway pressure; ΔP - driving pressure; MP - mechanical power; Crs - respiratory system compliance; ARDS - acute respiratory distress syndrome.

Figure 2S - Cumulative distribution of ventilation parameters.

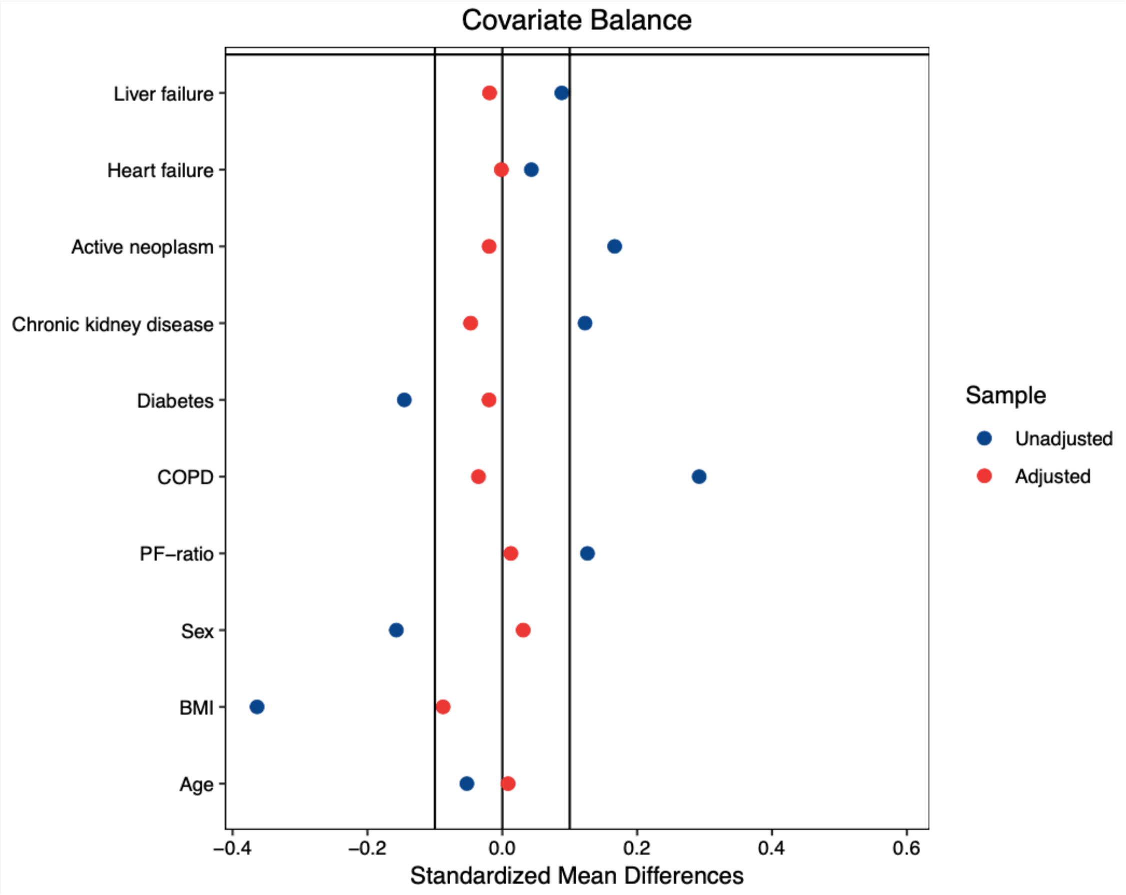

COPD - chronic obstructive pulmonary disease; PF - PaO_2/FiO_2 ratio; PaO_2 - partial pressure of arterial oxygen; FiO_2 - fraction of inspired oxygen; BMI - body mass index.

Figure 3S - Love plot propensity score matching.

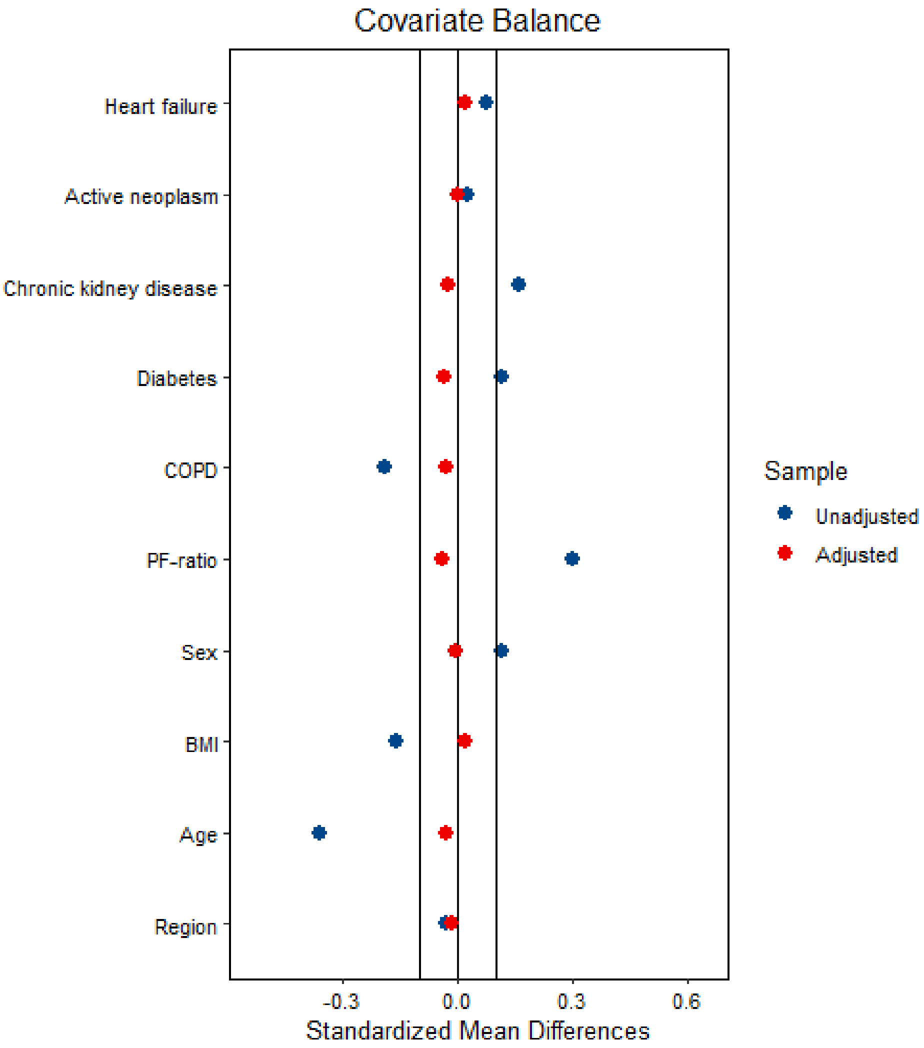

COPD - chronic obstructive pulmonary disease; PF- PaO_2/FiO_2 ratio; PaO_2 - partial pressure of arterial oxygen; FiO_2 - fraction of inspired oxygen; BMI - body mass index.

Figure 4S - Love plot propensity score matching for region.

LIST OF INVESTIGATORS FROM THE INCLUDED STUDIES

ERICC study

Amadeu Martinez, Livia Leal, Antonio Jorge Pereira, Marcelo de Oliveira Maia, Josè Aires Neto, Claudio Piras, Eliana Bernadete Caser, Cora Lavigne Moreira, Pablo Braga Gusman, Dyanne Moysés Dalcomune, Alexandre Guilherme Ribeiro de Carvalho, Louise Aline Romão Gondim, Lívia Mariane Castelo Branco Reis, Daniel da Cunha Ribeiro, Leonardo de Assis Simões, Rafaela Siqueira Campos, José Carlos Fernandez Versiani dos Anjos, Frederico Bruzzi Carvalho, Rossine Ambrosio Alves, Lilian Batista Nunes, Álvaro Réa-Neto, Mirella Cristine de Oliveira, Luana Tannous, Brenno Cardoso Gomes, Fernando Borges Rodriguez, Priscila Abelha, Marcelo E Lugarinho, Andre Japiassu, Hélder Konrad de Melo, Elton Afonso Lopes, Pedro Varaschin, Vicente Cés de Souza Dantas, Marcos Freitas Knibel, Micheli Ponte, Pedro Mendes de Azambuja Rodrigues, Rubens Carmo Costa Filho, Felipe Saddy, Théia Forny Wanderley Castellões, Suzana Alves Silva, Luiz Antonio Gomes Osorio, Dora Mannarino, Rodolfo Espinoza, Cassia Righy, Marcio Soares, Jorge Salluh, Lilian Tanaka, Daniel Aragão, Maria Eduarda Tavares, Maura Goncalves Pereira Kehdi, Valéria Maria Campos Rezende, Roberto Carlos Cruz Carbonell, Cassiano Teixeira, Roselaine Pinheiro de Oliveira, Juçara Gasparetto Maccari, Priscylla Souza Castro, Paula Berto, Patricia Schwarz, André Peretti Torelly, Thiago Lisboa, Edison Moraes, Felipe Dal-Pizzol, Cristiane Tomasi Damiani, Cristiane Ritter, Juliana Carvalho Ferreira, Ramon Teixeira Costa, Pedro Caruso, Cristina Prata Amendola, Amanda Maria R R de Oliveira, Ulysses V A Silva, Luciana Coelho Sanches, Rosana D S Almeida, Luciano Cesar Azevedo, Marcelo Park, Guilherme Schettino, Murillo Santucci Assunção, Eliezer Silva, Carlos Eduardo Barboza, Antonio Paulo Nassar Junior, Paulo Fernando G M Marzocchi Tierno, Luis Marcelo Malbouisson, Lucas Oliveira, Davi Cristovao, Manoel Leitão Neto, Ênio Rego, Fernanda Eugênia Fernandes, Marcelo Luz Pereira Romano, Alexandre Biasi Cavalcanti, Dalton de Souza Barros, Érica Aranha Suzumura, Karla Loureiro Meira, Gustavo Affonso de Oliveira, Paula Menezes Luciano, Evelin Drociunas Pacheco, Bruno Franco Mazza, Flavia Ribeiro Machado, Elaine Ferreira, Ronaldo Batista dos Santos, Alexandra Siqueira Colombo, Antonio Carlos Nogueira, Juliana Baroni Fernandes, Raquel Siqueira Nóbrega, Barbara do CS Martins, Francisco Soriano, Rafaela Deczka Morsch, Andre Luiz Baptiston Nunes, Juliano Pinheiro de Almeida, Ludhmila Hajjar, Sílvia Moulin, Fábio Poianas Giannini, and Andre Luiz Baptiston Nunes

LUNG SAFE

Fernando Rios, Frank Van Haren, T Sottiaux, Fredy S Lora, Luciano C Azevedo, P Depuydt, Eddy Fan, Guillermo Bugedo, Haibo Qiu, Marcos Gonzalez, Juan Silesky, Vladimir Cerny, Jonas Nielsen, Manuel Jibaja, Tài Pham, Hermann Wrigge, Dimitrios Matamis, Jorge Luis Ranero, S M Hashemian, Pravin Amin, Kevin Clarkson, Giacomo Bellani, Kiyoyasu Kurahashi, Asisclo Villagomez, Amine Ali Zeggwagh, Leo M Heunks, Jon Henrik Laake, Jose Emmanuel Palo, Antero do Vale Fernandes, Dorel Sandesc, Yaasen Arabi, Vesna Bumbasierevic, Jose A Lorente, Anders Larsson, Lise Piquilloud, Fekri Abroug, Daniel F McAuley, Lia McNamee, Javier Hurtado, Ed Bajwa, Gabriel Démpaire, Guy M Francois, Hektor Sula, Lordian Nunci, Alma Cani, Alan Zazu, Christian Dellera, Carolina S Insaurralde, Risso V Alejandro, Julio Daldin, Mauricio Vinzio, Ruben O Fernandez, Luis P Cardonnet, Lisandro R Bettini, Mariano Carboni Bisso, Emilio M Osman, Mariano G Setten, Pablo Lovazzano, Javier Alvarez, Veronica Villar, Cesar Milstein, Norberto C Pozo, Nicolas Grubissich, Gustavo A Plotnikow, Daniela N Vasquez, Santiago Ilutovich, Norberto Tiribelli, Ariel Chena, Carlos A Pellegrini, María G Saenz, Elisa Estenssoro, Matias Brizuela, Hernan Gianinetto, Pablo E Gomez, Valeria I Cerrato, Marco G Bezzi, Silvina A Borello, Flavia A Loiacono, Adriana M Fernandez, Serena Knowles, Claire Reynolds, Deborah M Inskip, Jennene J Miller, Jing Kong, Christina Whitehead, Shailesh Bihari, Aylin Seven, Amanda Krstevski, Helen J Rodgers, Rebecca T Millar, Toni E Mckenna, Irene M Bailey, Gabrielle C Hanlon, Anders Aneman, Joan M Lynch, Raman Azad, John Neal, Paul W Woods, Brigit L Roberts, Mark R Kol, Helen S Wong, Katharina C Riss, Thomas Staudinger, Xavier Wittebole, Caroline Berghe, Pierre A Bulpa, Alain M Dive, Rik Verstraete, Herve Lebbinck, Pieter Depuydt, Joris Vermassen, Philippe Meersseman, Helga Ceunen, Jonas I Rosa, Daniel O Beraldo, Claudio Piras, Adenilton M R Ampinelli, Antonio P Nassar Jr, Sergio Mataloun, Marcelo Moock, Marlus M Thompson, Claudio H Gonçalves, Ana Carolina P Antônio, Aline Ascoli, Rodrigo S Biondi, Danielle C Fontenele, Danielle Nobrega, Vanessa M Sales, Suresh Shindhe, Dk Maizatul Aiman B Pg Hj Ismail, John Laffey, Francois Beloncle, Kyle G Davies, Rob Cirone, Venika Manoharan, Mehvish Ismail, Ewan C Goligher, Mandeep Jassal, Erin Nishikawa, Areej Javeed, Gerard Curley, Nuttapol Rittayamai, Matteo Parotto, Niall D Ferguson, Sangeeta Mehta, Jenny Knoll, Antoine Pronovost, Sergio Canestrini, Alejandro R Bruhn, Patricio

H Garcia, Felipe A Aliaga, Pamela A Farías, Jacob S Yumha, Claudia A Ortiz, Javier E Salas, Alejandro A Saez, Luis D Vega, Eduardo F Labarca, Felipe T Martinez, Nicolás G Carreño, Pilar Lora, Haitao Liu, Haibo Qiu, Ling Liu, Rui Tang, Xiaoming Luo, Youzhong An, Huiying Zhao, Yan Gao, Zhe Zhai, Zheng L Ye, Wei Wang, Wenwen Li, Qingdong Li, Ruiqiang Zheng, Wenkui Yu, Juanhong Shen, Xinyu Li, Tao Yu, Weihua Lu, Ya Q Wu, Xiao B Huang, Zhenyang He, Yuanhua Lu, Hui Han, Fan Zhang, Renhua Sun, Hua X Wang, Shu H Qin, Bao H Zhu, Jun Zhao, Jian Liu, Bin Li, Jing L Liu, Fa C Zhou, Qiong J Li, Xing Y Zhang, Zhou Li-Xin, Qiang Xin-Hua, Liangyan Jiang, Yuan N Gao, Xian Y Zhao, Yuan Y Li, Xiao L Li, Chunting Wang, Qingchun Yao, Rongguo Yu, Kai Chen, Huanzhang Shao, Bingyu Qin, Qing Q Huang, Wei H Zhu, Ai Y Hang, Ma X Hua, Yimin Li, Yonghao Xu, Yu D Di, Long L Ling, Tie H Qin, Shou H Wang, Junping Qin, Yi Han, Suming Zhou, Monica P Vargas, Juan I Silesky Jimenez, Manuel A González Rojas, Jaime E Solis-Quesada, Christian M Ramirez-Alfaro, Jan Máca, Peter Sklienka, Jakob Gjedsted, Aage Christiansen, Jonas Nielsen, Boris G Villamagua, Miguel Llano, Philippe Burtin, Gautier Buzancais, Pascal Beuret, Nicolas Pelletier, Satar Mortaza, Alain Mercat, Jonathan Chelly, Sébastien Jochmans, Nicolas Terzi, Cédric Daubin, Guillaume Carteaux, Nicolas de Prost, Jean-Daniel Chiche, Fabrice Daviaud, Tai Pham, Muriel Fartoukh, Guillaume Barberet, Jerome Biehler, Jean Dellamonica, Denis Doyen, Jean-Michel Arnal, Anais Briquet, Sami Hraiech, Laurent Papazian, Arnaud Follin, Damien Roux, Jonathan Messika, Evangelos Kalaitzis, Laurence Dangers, Alain Combes, Siu-Ming Au, Gaetan Béduneau, Dorothée Carpentier, Elie H Zogheib, Herve Dupont, Sylvie Ricome, Francesco L Santoli, Sebastien L Besset, Philippe Michel, Bruno Gelée, Pierre-Eric Danin, Bernard Goubaux, Philippe J Crova, Nga T Phan, Frantz Berkelmans, Julio C Badie, Romain Tapponnier, Josette Gally, Samy Khebbeb, Jean-Etienne Herbrecht, Francis Schneider, Pierre-Louis M Declercq, Jean-Philippe Rigaud, Jacques Duranteau, Anatole Harrois, Russell Chabanne, Julien Marin, Charlene Bigot, Sandrine Thibault, Mohammed Ghazi, Messabi Boukhazna, Salem Ould Zein, Jack R Richecoeur, Daniele M Combaux, Fabien Grelon, Charlene Le Moal, Elise P Sauvadet, Adrien Robine, Virginie Lemiale, Danielle Reuter, Martin Dres, Alexandre Demoule, Dany Goldgran-Toledano, Loredana Baboi, Claude Guérin, Ralph Lohner, Jens Kraßler, Susanne Schäfer, Kai D Zacharowski, Patrick Meybohm, Andreas W Reske, Philipp Simon, Hans-Bernd F Hopf, Michael Schuetz, Thomas Baltus, Metaxia N Papanikolaou, Theonymfi G Papavasilopoulou, Giannis A Zacharas, Vasilis Ourailogloy, Eleni K Mouloudi, Eleni V Massa, Eva O Nagy, Electra E Stamou, Ellada V Kiourtzieva, Marina A Oikonomou, Luis E Avila, Cesar A Cortez, Johanna E Citalán, Sameer A Jog, Safal D Sable, Bhagyesh Shah, Mohan Gurjar, Arvind K Baronia, Mohammedfaruk Memon, Radhakrishnan Muthuchellappan, Venkatapura J Ramesh, Anitha Shenoy, Ramesh Unnikrishnan, Subhal B Dixit, Rachana V Rhayakar, Nagarajan Ramakrishnan, Vallish K Bhardwaj, Heera L Mahto, Sudha V Sagar, Vijayanand Palaniswamy, Deeban Ganesan, Seyed Mohammadreza Hashemian, Hamidreza Jamaati, Farshad Heidari, Edel A Meaney, Alistair Nichol, Karl M Knapman, Donall O'Croinin, Eimhin S Dunne, Dorothy M Breen, Kevin P Clarkson, Rola F Jaafar, Rory Dwyer, Fahd Amir, Olaitan O Ajetunmobi, Aogan C O'Muircheartaigh, Colin S Black, Nuala Treanor, Daniel V Collins, Wahid Altaf, Gianluca Zani, Maurizio Fusari, Savino Spadaro, Carlo A Volta, Romano Graziani, Barbara Brunettini, Salvatore Palmese, Paolo Formenti, Michele Umbrello, Andrea Lombardo, Elisabetta Pecci, Marco Botteri, Monica Savioli, Alessandro Protti, Alessia Mattei, Lorenzo Schiavoni, Andrea Tinnirello, Manuel Todeschini, Antonino Giarratano, Andrea Cortegiani, Sara Sher, Anna Rossi, Massimo M Antonelli, Luca M Montini, Paolo Casalena, Sergio Scafetti, Giovanna Panarello, Giovanna Occhipinti, Nicolò Patroniti, Matteo Pozzi, Roberto R Biscione, Michela M Poli, Ferdinando Raimondi, Daniela Albiero, Giulia Crapelli, Eduardo Beck, Vincenzo Pota, Vincenzo Schiavone, Alexandre Molin, Fabio Tarantino, Giacomo Monti, Elena Frati, Lucia Mirabella, Gilda Cinnella, Tommaso Fossali, Riccardo Colombo, Pierpaolo Terragni, Ilaria Pattarino, Francesco Mojoli, Antonio Braschi, Erika E Borotto, Andrea N Cracchiolo, Daniela M Palma, Francesco Raponi, Giuseppe Foti, Ettore R Vascotto, Andrea Coppadoro, Luca Brazzi, Leda Floris, Giorgio A Iotti, Aaron Venti, Osamu Yamaguchi, Shunsuke Takagi, Hiroki N Maeyama, Eizo Watanabe, Yoshihiro Yamaji, Kazuyoshi Shimizu, Kyoko Shiozaki, Satoru Futami, Sekine Ryosuke, Koji Saito, Yoshinobu Kameyama, Keiko Ueno, Masayo Izawa, Nao Okuda, Hiroyuki Suzuki, Tomofumi Harasawa, Michitaka Nasu, Tadaaki Takada, Fumihito Ito, Shin Nunomiya, Kansuke Koyama, Toshikazu Abe, Kohkichi Andoh, Kohei Kusumoto, Akira Hirata, Akihiro Takaba, Hiroyasu Kimura, Shuhei Matsumoto, Ushio Higashijima, Hiroyuki Honda, Nobumasa Aoki, Hiroshi Imai, Yasuaki Ogino, Ichiko Mizuguchi, Kazuya Ichikado, Kenichi Nitta, Katsunori Mochizuki, Tomoaki Hashida, Hiroyuki Tanaka, Tomoyuki Nakamura, Daisuke Niimi, Takeshi Ueda, Yozo Kashiwa, Akinori Uchiyama, Olegs Sabelnikovs, Peteris Oss, Youssef Haddad, Kong Y Liew,

Silvio A Ñamendys-Silva, Yves D Jarquin-Badiola, Luis A Sanchez-Hurtado, Saira S Gomez-Flores, Maria C Marin, Asisclo J Villagomez, Jordana S Lemus, Jonathan M Fierro, Mavy Ramirez Cervantes, Francisco Javier Flores Mejia, Daniel R Gonzalez, Dulce M Dector, Claudia R Estrella, Jorge R Sanchez-Medina, Alvaro Ramirez-Gutierrez, Fernando G George, Janet S Aguirre, Juan A Buensuseso, Manuel Poblano, Tarek Dendane, Amine Ali Zeggwagh, Hicham Balkhi, Mina Elkhayari, Nacer Samkaoui, Hanane Ezzouine, Abdellatif Benslama, Mourad Amor, Wajdi Maazouzi, Nedim Cimic, Oliver Beck, Monique M Bruns, Jeroen A Schouten, Myra Rinia, Monique Raaijmakers, Leo M Heunks, Hellen M Van Wezel, Serge J Heines, Marc P Buise, Fabienne D Simonis, Marcus J Schultz, Jennifer C Goodson, Troy S B rowne, Leanlove Navarra, Anna Hunt, Robyn A Hutchison, Mathew B Bailey, Lynette Newby, Colin Mcarthur, Michael Kalkoff, Alex Mcleod, Jonathan Casement, Danielle J Hacking, Finn H Andersen, Merete S Dolva, Jon H Laake, Andreas Barratt-Due, Kim Andre L Noremark, Eldar Søreide, Brit Å Sjøbø, Anne B Guttormsen, Hector H Leon Yoshido, Ronald Zumaran Aguilar, Fredy A Montes Oscanoa, Alain U Alisasis, Joanne B Robles, Rossini Abbie B Pasanting-Lim, Beatriz C Tan, Pawel Andruszkiewicz, Karina Jakubowska, Cristina M Cox, António M Alvarez, Bruno S Oliveira, Gustavo M Montanha, Nelson C Barros, Carlos S Pereira, António M Messias, Jorge M Monteiro, Ana M Araujo, Nuno T Catorze, Susan M Marum, Maria J Bouw, Rui M Gomes, Vania A Brito, Silvia Castro, Joana M Estilita, Filipa M Barros, Isabel M Serra, Aurelia M Martinho, Dana R Tomescu, Alexandra Marcu, Ovidiu H Bedreag, Marius Papurica, Dan E Corneci, Silvius Ioan Negoita, Evgeny Grigoriev, Alexey I Gritsan, Andrey A Gazenkampf, Ghaleb Almekhlafi, Mohamad M Albarrak, Ghanem M Mustafa, Khalid A Maghrabi, Nawal Salahuddin, Tharwat M Aisa, Ahmed S Al Jabbary, Edgardo Tabhan, Yaseen M Arabi, Olivia A Trinidad, Hasan M Al Dorzi, Edgardo E Tabhan, Stefan Bolon, Oliver Smith, Jordi Mancebo, Hernan Aguirre-Bermeo, Juan C Lopez-Delgado, Francisco Esteve, Gemma Rialp, Catalina Forteza, Candelaria De Haro, Antonio Artigas, Guillermo M Albaiceta, Sara De Cima-Iglesias, Leticia Seoane-Quiroga, Alexandra Ceniceros-Barros, Antonio L Ruiz-Aguilar, Luis M Claraco-Vega, Juan Alfonso Soler, Maria del Carmen Lorente, Cecilia Hermosa, Federico Gordo, Miryam Prieto-González, Juan B López-Messa, Manuel P Perez, Cesar P Pere, Raquel Montoiro Allue, Ferran Roche-Campo, Marcos Ibañez-Santacruz, Susana Temprano, Maria C Pintado, Raul De Pablo, Pilar Ricart Aroa Gómez, Silvia Rodriguez Ruiz, Silvia Iglesias Moles, Mª Teresa Jurado, Alfons Arizmendi, Enrique A Piacentini, Nieves Franco, Teresa Honrubia, Meisy Perez Cheng, Elena Perez Losada, Javier Blanco, Luis J Yuste, Cecilia Carbayo-Gorriz, Francisca G Cazorla-Barranquero, Javier G Alonso, Rosa S Alda, Ángela Algaba, Gonzalo Navarro, Enrique Cereijo, Esther Diaz-Rodriguez, Diego Pastor Marcos, Laura Alvarez Montero, Luis Herrera Para, Roberto Jimenez Sanchez, Miguel Angel Blasco Navalpotro, Ricardo Diaz Abad, Raquel Montiel González, Dácil Parrilla Toribio, Alejandro G Castro, Maria Jose D Artiga, Oscar Penuelas, Tomas P Roser, Moreno F Olga, Elena Gallego Curto, Rocío Manzano Sánchez, Vallverdu P Imma, Garcia M Elisabet, Laura Claverias, Monica Magret, Ana M Pellicer, Lucia L Rodriguez, Jesús Sánchez-Ballesteros, Ángela González-Salamanca, Antonio G Jimenez, Francisco P Huerta, Juan Carlos J Sotillo Diaz, Esther Bermejo Lopez, David D Llinares Moya, Alec A Tallet Alfonso, Palazon Sanchez Eugenio Luis, Palazon Sanchez Cesar, Sánchez I Rafael, Corcoles G Virgilio, Noelia N Recio, Richard O Adamsson, Christian C Rylander, Bernhard Holzgraefe, Lars M Broman, Joanna Wessbergh, Linnea Persson, Fredrik Schiöler, Hans Kedelv, Anna Oscarsson Tibblin, Henrik Appelberg, Lars Hedlund, Johan Helleberg, Karin E Eriksson, Rita Glietsch, Niklas Larsson, Ingela Nygren, Silvia L Nunes, Anna-Karin Morin, Thomas Kander, Anne Adolfsson, Lise Piquilloud, Hervé O Zender, Corinne Leemann-Refondini, Souheil Elatrous, Slaheddine Bouchoucha, Imed Chouchene, Islem Ouanes, Asma Ben Souissi, Salma Kamoun, Oktay Demirkiran, Mustafa Aker, Emre Erbabacan, Ilkay Ceylan, Nermin Kelebek Girgin, Menekse Ozcelik, Necmettin Ünal, Basak Ceyda Meco, Onat O Akyol, Suleyman S Derman, Barry Kennedy, Ken Parhar, Latha Srinivasa, Lia McNamee, Danny McAuley, Jack Steinberg, Phil Hopkins, Clare Mellis, Frank Stansil, Vivek Kakar, Dan Hadfield, Christine Brown, Andre Vercueil, Kaushik Bhowmick, Sally K Humphreys, Andrew Ferguson, Raymond Mckee, Ashok S Raj, Danielle A Fawkes, Philip Watt, Linda Twohey, Rajeev R Jha Matthew Thomas, Alex Morton, Varsha Kadaba, Mark J Smith, Anil P Hormis, Santhana G Kannan, Miriam Namih, Henrik Reschreiter, Julie Camsooksai, Alek Kumar, Szabolcs Rugonfalvi, Christopher Nutt, Orla Oneill, Colette Seasman, Ged Dempsey, Christopher J Scott, Helen E Ellis, Stuart Mckechnie, Paula J Hutton, Nora N Di Tomasso, Michela N Vitale, Ruth O Griffin, Michael N Dean, Julius H Cranshaw, Emma L Willett, Nicholas Ioannou, Sarah Gillis, Peter Csabi, Rosaleen Macfadyen, Heidi Dawson, Pieter D Preez, Alexandra J Williams, Owen Boyd, Laura Ortiz-Ruiz De Gordoa, Jon Bramall, Sophie Symmonds, Simon K Chau, Tim Wenham, Tamas Szakmany, Piroska

Toth-Tarsoly, Katie H Mccalman, Peter Alexander, Lorraine Stephenson, Thomas Collyer, Rhiannon Chapman, Raphael Cooper, Russell M Allan, Malcolm Sim, David W Wrathall, Donald A Irvine, Kim S Zantua, John C Adams, Andrew J Burtenshaw, Gareth P Sellors, Ingeborg D Welters, Karen E Williams, Robert J Hessell, Matthew G Oldroyd, Ceri E Battle, Suresh Pillai, Istvan Kajtor, Mageswaran Sivashanmugave, Sinead C Okane, Adrian Donnelly, Aniko D Frigyik, Jon P Careless, Martin M May, Richard Stewart, T John Trinder, Samantha J Hagan, Matt P Wise, Jade M Cole, Caroline C MacFie, Anna T Dowling, Javier Hurtado, Nicolás Nin, Javier Hurtado, Edgardo Nuñez, Gustavo Pittini, Ruben Rodriguez, María C Imperio, Cristina Santos, Ana G França, Alejandro Ebeid, Alberto Deicas, Carolina Serra, Aditya Uppalapati, Ghassan Kamel, Valerie M Banner-Goodspeed, Jeremy R Beitler, Satyanarayana Reddy Mukkera, Shreedhar Kulkarni, Jarone Lee, Tomaz Mesar, John O Shinn Iii, Dina Gomaa, Christopher Tainter, Tomaz Mesar, R Adams Cowley, Dale J Yeatts, Jessica Warren, Michael J Lanspa, Russel R Miller, Colin K Grissom, Samuel M Brown, Philippe R Bauer, Ryan J Gosselin, Barrett T Kitch, Jason E Cohen, Scott H Beegle, Renaud M Gueret, Aiman Tulaimat, Shazia Choudry, William Stigler, Hitesh Batra, Nidhi G Huff, Keith D Lamb, Trevor W Oetting, Nicholas M Mohr, Claine Judy, Shigeki Saito, Fayez M Kheir, Adam B Schlichting, Angela Delsing, Mary Elmasri, Daniel R Crouch, Dina Ismail, Thomas C Blakeman, Kyle R Dreyer, Dina Gomaa, Rebecca M Baron, Carolina Quintana Grijalba, Peter C Hou, Raghu Seethala, Imo Aisiku, Galen Henderson, Gyorgy Frendl, Sen-Kuang Hou, Robert L Owens, Ashley Schomer, Vesna Bumbasirevic, Bojan Jovanovic, Maja Surbatovic, and Milic Veljovic

PRoVENT–COVID

Jesse P. van Akkeren, Anna Geke Algera, Cheetel K. Algoe, Rombout B. van Amstel, Onno L. Baur, Pablo van de Berg, Alida E. van den Berg, Dennis C.J.J. Bergmans, Dido I. van den Bersselaar, Freke A. Bertens, Alexander J.G.H. Bindels, Siebe G. Blok, Milou M. de Boer, Sylvia den Boer, Leonoor S. Boers, Margriet Bogerd, Lieuwe D.J. Bos, Michela Botta, Jennifer S. Breel, Hendrik de Bruin, Sanne de Bruin, Caro L. Bruna, Laura A. Buiteman-Kruizinga, Olaf L. Cremer, Rogier M. Determann, Willem Dieperink, Dave A. Dongelmans, Hildegard S. Franke, Michal S. Galek-Aldridge, Mart J. de Graaff, Laura A. Hagens, Jasper J. Haringman, Sebastiaan T. van der Heide, Pim L.J. van der Heiden, Nanon F.L. Heijnen, Stephan J.P. Hiel, Lotte L. Hoeijmakers, Liselotte Hol, Markus W. Hollmann, Marga E. Hoogendoorn, Janneke Horn, Robrecht van der Horst, Evy L.K. Ie, Dimitri P. Ivanov, Nicole Juffermans, Eline Kho, Eline S. de Klerk, Ankie W.M.M. Koopman-van Gemert, Matty Koopmans, Songul Kucukcelebi, Michael A. Kuiper, Dylan W. de Lange, Niels van Mourik, Sunny G.L.H. Nijbroek, Marisa Onrust, Evelien A.N. Oostdijk, Frederique Paulus, Charlotte J. Pennartz, Janesh Pillay, Luigi Pisani, Ilse M. Purmer, Thijs C.D. Rettig, Jan-Paul Roozeman, Michiel T.U. Schuijt, Marcus J. Schultz, Ary Serpa Neto, Mengalvio E. Sleeswijk, Marry R. Smit, Peter E. Spronk, Willemke Stilma, Aart C. Strang, Anissa M. Tsonas, Pieter R. Tuinman, Christel M.A. Valk, Felicia L. Veen-Schra, Lars I. Veldhuis, Patricia van Velzen, Fleur L.I.M. van der Ven, Ward H. van der Ven, Alexander P.J. Vlaar, Peter van Vliet, Peter H.J. van der Voort, Louis van Welie, Henrico J.F.T. Wesselink, Hermien H. van der Wier-Lubbers, Bas van Wijk, Tineke Winters, Wing Yi Wong, and Arthur R.H. van Zanten

EPICCoV

Juliana C. Ferreira, Yeh-Li Ho, Bruno A.M.P. Besen, Luiz M.S. Malbuisson, Leandro U. Taniguchi,IV Pedro, Mendes, Eduardo L.V. Costa, Marcelo Park, Renato Daltro-Oliveira, Roberta M.L. Roepke, João M. Silva, Jr, Maria José C. Carmona, Carlos Roberto Ribeiro Carvalho, Adriana Hirota, Alberto Kendy Kanasiro, Alessandra Crescenzi, Amanda Coelho Fernandes, Anna Miethke-Morais, Arthur Petrillo Bellintani, Artur Ribeiro Canasiro, Bárbara Vieira Carneiro, Beatriz Keiko Zanbon, Bernardo Pinheiro De Senna Nogueira Batista, Bianca Ruiz Nicolao, Bruno Adler Maccagnan Pinheiro Besen, Bruno Biselli, Bruno Rocha De Macedo, Caio Machado Gomes De Toledo, Carlos Eduardo Pompilio, Carlos Roberto Ribeiro De Carvalho, Caroline Gomes Mol, Cassio Stipanich, Caue Gasparotto Bueno, Cibele Garzillo, Clarice Tanaka, Daniel Neves Forte, Daniel Joelsons, Daniele Robira, Eduardo Leite Vieira Costa, Elson Mendes Da Silva, Júnior, Fabiane Aliotti Regalio, Gabriela Cardoso Segura, Gustavo Brasil Marcelino, Giulia Sefrin Louro, Yeh-Li Ho, Isabela Argollo Ferreira, Jeison de Oliveira Gois, Joao Manoel Da Silva, Junior, Jose Otto Reusing, Junior, Julia Fray Ribeiro, Juliana Carvalho Ferreira, Karine Vusberg Galleti, Katia Regina Silva, Larissa Padrao Isensee, Larissa dos Santos Oliveira, Leandro Utino Taniguchi, Leila Suemi Letaif, Lígia Trombetta Lima, Lucas Yongsoo Park, Lucas Chaves, Netto, Luciana Cassimiro Nobrega, Luciana Haddad, Ludhmila Hajjar, Luiz Marcelo Malbouisson, Manuela Cristina Adsuara Pandolfi, Marcelo Park, Maria José Carvalho Carmona, Maria Castilho Prandini H

De Andrade, Mariana Moreira Santos, Matheus Pereira Bateloche, Mayra Akimi Suiama, Mayron Faria de Oliveira, Mayson Laercio Sousa, Michelle Louvaes, Natassja Huemer, Pedro Mendes, Paulo Ricardo Gessolo Lins, Pedro Gaspar Dos Santos, Pedro Ferreira Paiva Moreira, Renata Mello Guazzelli, Renato Batista Dos Reis, Renato Daltro De Oliveira, Roberta Muriel Longo Roepke, Rodolpho Augusto De Moura Pedro, Rodrigo Kondo, Samia Zahi Rached, Sergio Roberto Silveira Da Fonseca, Thais Sousa Borges, Thalissa Ferreira, Vilson Cobello, Junior, Vivian Vieira Tenório Sales, and Willaby Serafim Cassa Ferreira

CIBERESUCICOVID

Rafael Mañez, Felipe Rodríguez de Castro, María Mora Aznar, Mateu Torres, María Martinez, Cynthia Alegre, Sofía Contreras, Javier Trujillano, Montse Vallverdú, Miguel León, Mariona Badía, Begoña Balsera, Lluís Servià, Judit Vilanova, Silvia Rodríguez, Neus Montserrat, Silvia Iglesias, Javier Prados, Sula Carvalho, Mar Miralbés, Josman Monclou, Gabriel Jiménez, Jordi Codina, Estela Val, Pablo Pagliarani, Jorge Rubio, Dulce Morales, Andrés Pujol, Àngels Furro, Beatriz García, Gerard Torres, Javier Vengoechea, David de Gozalo Calvo, Jessica González, Silvia Gomez, Lorena Forcelledo Espina, Emilio García Prieto, Paula Martín Vicente, Cecilia del Busto Martínez, María Aguilar Cabello, Carmen Eulalia Martínez Fernández, María Luisa Blasco Cortés, Ainhoa Serrano Lázaro, Mar Juan Díaz, María Teresa Bouza Vieiro, Inés Esmorís Arijón, David Campi Hermoso, Rafaela Nogueras Salinas., Teresa Farre Monjo., Ramon Nogue Bou., Gregorio Marco Naya., Núria Ramon Coll, Juan Carlos Montejo-González, Gloria Renedo Sanchez-Giron, Juan Bustamante-Munguira, Ramon Cicuendez Avila, Nuria Mamolar Herrera, Alexander Agrifoglio, Lucia Cachafeiro, Emilio Maseda, Albert Figueras, Maria Teresa Janer, Laura Soliva, Marta Ocón, Luisa Clar, J Ignacio Ayestarán, Sandra Campos Fernández, Eva Forcadell-Ferreres, Immaculada Salvador-Adell, Neus Bofill, Berta Adell-Serrano, Josep Pedregosa Díaz, Núria Casacuberta-Barberà, Luis Urrelo-Cerrón, Àngels Piñol-Tena, Ferran Roche-Campo, Pablo Ryan Murúa, Covadonga Rodríguez Ruíz, Laura Carrión García, Juan I Lazo Álvarez, Desire Macias Guerrero, Daniel Tognetti, Carlos García Redruello, David Mosquera Rodríguez, Eva María Menor Fernández, Sabela Vara Adrio, Vanesa Gómez Casal, Marta Segura Pensado, María Digna Rivas Vilas, Amaia García Sagastume, Raul de Pablo Sánchez, David Pestaña Laguna, Tommaso Bardi, Carmen Gómez Gonzalez, Maria Luisa Gascón Castillo, José Garnacho-Montero, Joan Ramon Masclans, Ana Salazar Degracia, Judit Bigas, Rosana Muñoz-Bermúdez, Clara Vilà-Vilardel, Francisco Parrilla, Irene Dot, Ana Zapatero, Yolanda Díaz, María Pilar Gracia, Purificación Pérez, Andrea Castellví, Cristina Climent, Lidia Serra, Laura Barbena, Iosune Cano, Alba Herraiz, Pilar Marcos, Laura Rodríguez, Maria Teresa Sariñena, Ana Sánchez, Juan Fernando Masa Jimenez, Gemma Gomà, Mercedes Ibarz, Diego De Mendoza, Enric Barbeta, Victoria Alcaraz-Serrano, Joan Ramon Badia, Manuel Castella, Leticia Bueno, Laia Fernandez Barat, Catia Cillóniz, Pamela Conde, Javier Fernández, Albert Gabarrus, Karsa Kiarostami, Alexandre López-Gavín, Cecilia L Mantellini, Carla Speziale, Nil Vázquez, Hua Yang, Minlan Yang, Carlos Ferrando, Pedro Castro, Marta Arrieta, Jose Maria Nicolas, Rut Andrea, Marta Barroso, Sergio Álvarez, Dario Garcia-Gasulla, Adrián Tormos, Cesar Aldecoa, Rubén Herrán-Monge, José Ángel Berezo García, Pedro Enríquez Giraudo, Pablo Cardinal Fernández, Alberto Rubio López, Orville Báez Pravia, Leire Pérez Bastida, Antonjo Alvarez Ruiz, Anna Parera Pous, Ana López Lago, Eva Saborido Paz, Patricia Barral Segade, Manuel Valledor Mendez, Luciano Aguilera, Esther López-Ramos, Ángela Leonor Ruiz-García, Belén Beteré, Rafael Blancas, Cristina Dólera, Gloria Perez Planelles, Enrique Marmol Peis, Maria Dolores Martinez Juan, Miriam Ruiz Miralles, Eva Perez Rubio, Maria Van der Hofstadt Martin-Montalvo, Tatiana Villada Warrington, Sara Guadalupe Moreno Cano, Federico Gordo, Basilisa Martinez Palacios, Maria Teresa Nieto, Sergio Ossa, Ana Ortega, Miguel Sanchez, Bitor Santacoloma.

SATI–COVID-19

Elisa Estenssoro, Arnaldo Dubin, Cecilia Inés Loudet, Fernando Ríos, Vanina Siham Kanoore Edul, Gustavo Plotnikow, Rosa Reina Macarena Andrian, Julián Ivacachi, Ignacio Romero, Carla Garay, Damián Piezny, Judith Sagardía, Marco Bezzi, Silvia Borello, Verónica Mandich, Daniel Chiacchiara, Carla Groer, Constanza García Almirón, Ana Kovac, Sebastián Torres, Cristian Cesio, Cristina Orlandi, Rosana Hernández, Paolo Nahuel Rubatto Birri, Matías Mugno, Florencia Valenti, Raúl Alejandro Gómez, Eleonora Cunto, Viviana Chediack, María Gabriela Sáenz, Cecilia Marchena, Norberto Tiribelli, María Guayma, Vanina Aphalo, Daniela Vazquez, Yasmin Saad, Diego Sanchez, Federico Iglesias, Pablo Casteluccio, Bernardo Lattanzio, Sebastián Eiguren, Diego Noval, Sebastián Fredes, Gabriela Izzo, Horacio Cabrera, Mario Pozo, Santiago Sac, Nicolás Tornatore, Julia Sakugawa, Celeste Villafañe, Antonio Di Sibio, Patricio Maskin, Pablo Rodríguez, Nicolás

Nihany, Mariela Mogadouro, Fernando Pálizas, Emiliano Cornú, Mariano Esperatti, Juan Manuel Pintos, Gustavo Badariotti, Gonzalo Echevarría, Ana María Mazzola, Cecilia Giuggia, Nahuel Dargains, Alejandra Turano, Florencia Pugliese, Marcos Zec Baskarad, Mariana Chamadoira, Juan Carlos Medina, Marina Búsico, Fernando Villarejo, Hugo Collazos, Tania Huanca, Juan Carlos Pendino, Lionel Talamonti, Fernando Skrzypiec, Claudia Tascón, Gabriela Genovese, Hugo Alul, Agustina Zavattieri, Ana Julieta Herrera, Norma Rosales, María Gabriela Quintana, Alejandro Risso Vazquez, Martín Lugaro, Eduardo Díaz Rousseaux, Marcelo Falcone, Fernando Kurban, Matías Cini, Graciela Zakalik, Carlos Pellegrini, Gabriela Fernández, Juan Pablo Sottile, Sol Barrios, Orlando Hamada, Verónica Mendiluce, Darío Villalba, Florencia Sacco , Vito Mezzina, Carlos Servin, Mónica Quinteros, Hernán Nuñez, María Luz Campassi, David Banegas, Carina Balasini, Victoria Leiva, Franco Maicol, Gustavo Domeniconi , Verónica Vilaseca, Alejandra Barrientos , Florencia Larocca, Liliana Kumar, Rosa Luna, Martín Deheza Lonardi, Agustina Oholeguy, Joaquín Carnero Echegaray, Carla Marazzi, Plácido Helca Regis, Federico Rópolo, Adrián Bobadilla, Vivian Thomas, Nydia Funes Nelson, Cintia Villavicencio, Pedro Machare, Norma Aramayo, Cecilia González, Mariano Ferriccioni, Judith Bergesio.
